# Genomic analyses of fairy and fulmar prions (Procellariidae: *Pachyptila* spp.) reveals parallel evolution of bill morphology, and multiple species

**DOI:** 10.1371/journal.pone.0275102

**Published:** 2022-09-27

**Authors:** Lara D. Shepherd, Colin M. Miskelly, Mariana Bulgarella, Alan J. D. Tennyson

**Affiliations:** Museum of New Zealand Te Papa Tongarewa, Wellington, New Zealand; MARE – Marine and Environmental Sciences Centre, PORTUGAL

## Abstract

Prions are small petrels that are abundant around the Southern Ocean. Here we use mitochondrial DNA (COI and cytochrome *b*) and nuclear reduced representation sequencing (ddRADseq) to examine the relationships within and between fairy (*Pachyptila turtur*) and fulmar (*P*. *crassirostris*) prions from across their distributions. We found that neither species was recovered as monophyletic, and that at least three species were represented. Furthermore, we detected several genetic lineages that are also morphologically distinct occurring in near sympatry at two locations (Snares Islands and Chatham Islands). The factors that have driven diversification in the fairy/fulmar prion complex are unclear but may include philopatry, differences in foraging distribution during breeding, differences in non-breeding distributions and breeding habitat characteristics. The observed distribution of genetic variation in the fairy/fulmar prion complex is consistent with population expansion from ice-free Last Glacial Maximum refugia into previously glaciated areas.

## Introduction

Prions (*Pachyptila*) are one of the most abundant groups of seabirds, with an estimated 95 million individuals [1]. These small petrels are mostly found in the Southern Ocean where they usually breed on remote, predator-free islands. Species limits within *Pachyptila* have long been debated [2–7] and have led to numerous names now in synonomy [8]. The morphological similarity between prion species, in particular the overlap in morphological measurements and colour patterns, has led to uncertainty regarding the number of species [1, 7, 9, 10]. All species are similar in appearance and behavior, differing mainly in the size and structure of their bills [11], which reflects differences in prey selection and feeding strategies. Six or seven species are currently recognised: Antarctic prion (*P*. *desolata* (Gmelin, 1789)), broad-billed prion (*P*. *vittata* (G. Forster, 1777)), fairy prion (*P*. *turtur* (Kuhl, 1820)), fulmar prion (*P*. *crassirostris* (Mathews, 1912)), Salvin’s prion (*P*. *salvini* (Mathews, 1912)), thin-billed prion (*P*. *belcheri* (Mathews, 1912)) and Macgillivray’s prion (*P*. *macgillivrayi* (Mathews, 1912)), which has alternatively been treated as a subspecies of either Salvin’s prion or broad-billed prion.

These species are closely-related, with molecular dating estimating that they diverged within the last 6 million years [12]. Genetic studies have clarified some of the relationships between prion species [7, 12–15]. For example, Salvin’s prion appears to be a hybrid between broad-billed prion and Antarctic prion [12]. However, questions remain.

The focus of our study is the fairy and fulmar prion species complex. Fairy and fulmar prions are morphologically very similar and are difficult to distinguish at sea [16]; they have been considered conspecific by some authors [5, 9]. Nevertheless, fairy prions have a more slender bill with a larger gap between the nostril tube and terminal nail than fulmar prions [17], and their calls also differ [16, 18].

**Fig 1:**
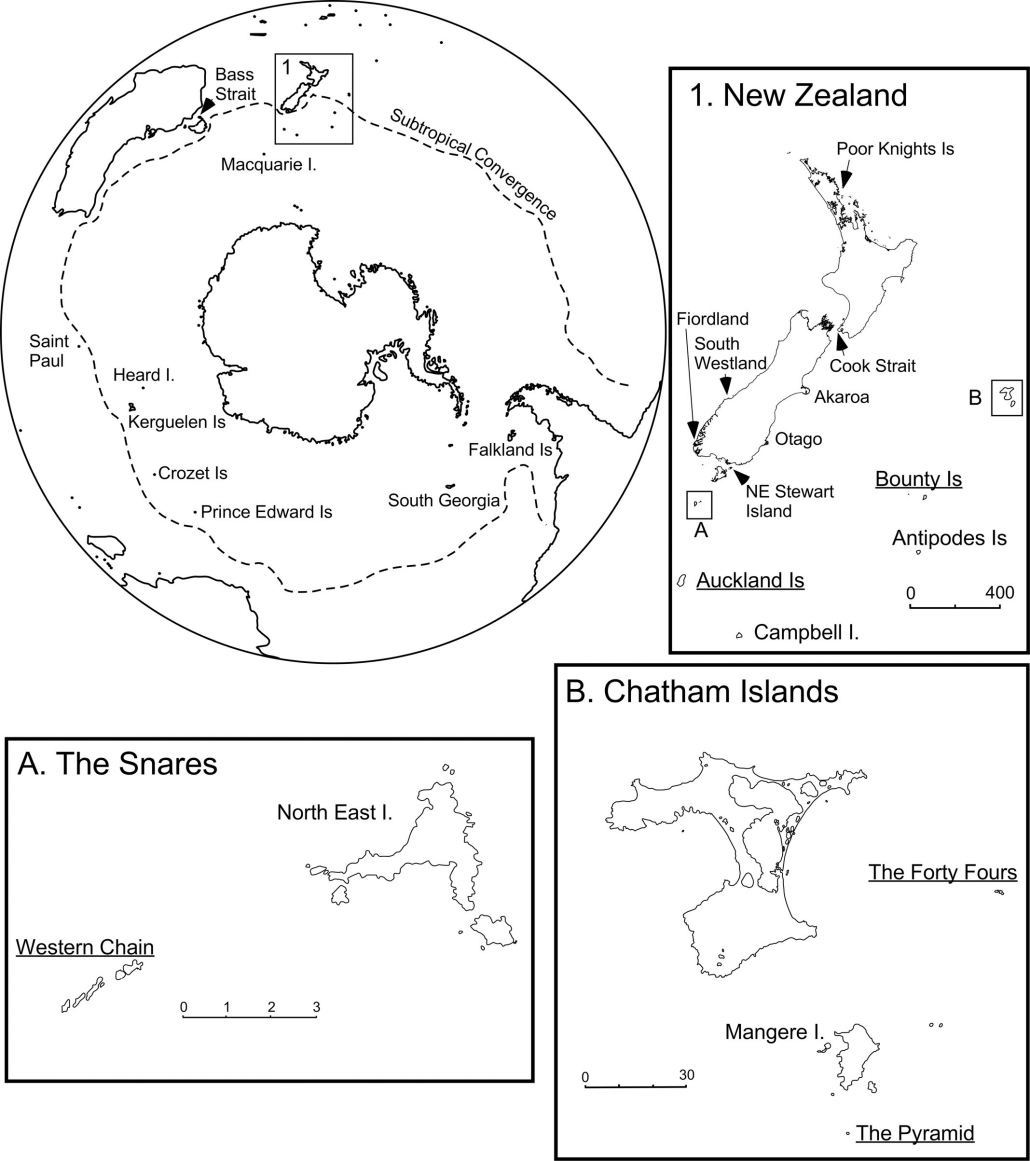
Distribution map of fairy (*Pachyptila turtur*) and fulmar (*Pachyptila crassirostris*) prion breeding colonies. The location names of fulmar prion breeding colonies are underlined and the location of fairy prion breeding colonies shown in bold and not underlined. Inset 1 includes the New Zealand region, insets A and B are within this region. Note that we here report *P*. *crassirostris* to be confined to the New Zealand region, with Heard Island birds identified as *P*. *turtur* (see Results).

Fairy and fulmar prions broadly overlap in distribution within the New Zealand region (Fig 1). Both species occur on the Chatham and Snares Islands; however, each breeds on different islands within these archipelagos (Fig 1). Fairy prions have a circumpolar distribution, breeding on numerous subantarctic and subtropical southern hemisphere islands (Fig 1). They are particularly common in New Zealand, where they are one of the most abundant breeding seabird species [19]. Some authors recognise two subspecies of fairy prion: *P*. *turtur subantarctica*, which breeds on the Antipodes Islands, Snares Islands and Macquarie Island and *P*. *turtur turtur*, which breeds on the remaining islands in this species’ range [6, 16] (Fig 1).

In contrast, fulmar prions have a much smaller distribution and only breed on remote islands (Fig 1). Three subspecies are currently recognised within fulmar prions [8]: *P*. *c*. *crassirostris* (Mathews, 1912), which nests on the Bounty Islands and Toru and Rima islets of the Western Chain, Snares Islands [18, 20]; *P*. *c*. *pyramidalis* C.A. Fleming, 1939, which breeds on The Pyramid and The Forty-Fours in the Chatham Islands [19]; and *P*. *c*. *flemingi* Tennyson & Bartle, 2005, which breeds on Ewing, Ocean, Disappointment, Monumental, and Rose Islands in the Auckland Islands [21]. This latter form was formerly considered to breed on Heard Island and possibly McDonald Island [22].

Several genetic studies have included fairy prion samples, but these had very limited geographical sampling [7, 13–15, 23], with at most three geographic regions sampled (Australia, mainland New Zealand and the Antipodes Islands [15]). Only one genetic study [15] has included fulmar prions with the single fulmar prion specimen they included nested within their six fairy prion specimens in their mitochondrial DNA (mtDNA) cytochrome *c* oxidase subunit I (COI) phylogeny.

In this study we examine the relationships within and between fairy and fulmar prion populations using mitochondrial DNA sequencing and high throughput sequencing of nuclear DNA (double-digest restriction site-associated DNA sequencing, ddRADseq; [24]). Previous studies of genetic variation within other seabird species have found varying levels of genetic partitioning ranging from strong genetic structuring to virtual panmixia (reviewed in [25]). Physical barriers, such as land or ice, often provide a significant restriction to gene flow in seabirds and have led to strong genetic structuring [26]. However, there are few contemporary physical barriers within the distributions of fairy and fulmar prions. Instead, factors such as ocean currents, at-sea distributions and past glaciation may influence their genetic patterns, as has been reported for other Southern Ocean seabirds [27]. Philopatry, which can lead to high levels of genetic structuring, has been inferred based on a mtDNA study of Australian fairy prion colonies [23], as well as from mark-recapture studies on Mana Island [28] and the Snares Islands (Tennyson pers. obs.).

Fairy and fulmar prions are both listed by the International Union for Conservation of Nature as of ‘Least Concern’ [29, 30]. However, they still face significant population threats, including vulnerability to introduced predators, storm impacts, climate change and decrease in ocean productivity (reviewed in [19]). Understanding the distribution of genetic variation and connectivity between colonies is important for tracking how threats are impacting fairy and fulmar prion populations.

## Material and methods

### Taxon sampling

Both modern samples and historical skins were included in this study (S1 and S2 Tables), representing most of the geographic spread of breeding colonies of fairy and fulmar prions. Modern samples from live or dead birds consisted of blood, feathers or skin tissue. Blood samples were collected under a permit from the Department of Conservation (permit number 97330-FLO). Sampling methods were carried out under approved ethical procedures as required under this permit.

Study skins were sampled from the collection of the Museum of New Zealand Te Papa Tongarewa, Wellington, New Zealand (NMNZ). The oldest of these was collected in 1929 (S2 Table). Tissue samples (footpad or skin surrounding existing damage to the specimen) were removed with a sterile razor blade. Two Holocene-aged fossil bones were also sampled by removing 0.5 mm from the broken ends with a Dremel grinder, using a new Dremel wheel for each bone.

### DNA extraction

Modern samples were extracted with either proteinase K digestion followed by phenol-chloroform extraction [31] or a DNeasy Blood and Tissue kit (Qiagen, Valencia, California, USA), following the manufacturer’s instructions.

DNA extractions and polymerase chain reaction (PCR) set-ups of historical specimens were performed in an ancient DNA (aDNA) laboratory, which is physically isolated from where modern DNA and PCR products are handled. Potential contamination was monitored by the use of negative extraction and PCR controls.

Tissue samples from museum study skins were extracted using a Qiagen DNeasy Blood and Tissue kit, following the manufacturer’s instructions except that the final elution used 50 μl of Buffer AE.

The bone samples were ground in a mortar and pestle, cleaned with bleach and UV-light between samples. The bone powder was then decalcified and a phenol-chloroform extraction performed [32].

### mtDNA sequencing

Primers [33] were used to amplify either the full length (modern samples) or overlapping fragments (historical samples) of the 648 base pair (bp) barcoding region of the mitochondrial cytochrome oxidase I (COI) locus. A 880 bp fragment of the mitochondrial cytochrome *b* gene was amplified from a subset of samples (only the modern and well-preserved historical samples) to provide greater resolution of phylogenetic relationships, using the CytB_Pri_F and CytB_Pri_R primers [13].

PCR amplification was performed in 12 μl volumes containing 1× MyTaq mix (Bioline, Australia), 0.3 M of BSA and 0.5 μM of each primer. For all PCR amplifications the thermocycling conditions involved an initial denaturation of 98°C for 1 minute then 35 cycles of 98°C for 20 seconds, 50°C for 20 seconds and 72°C for 30 seconds, followed by a final extension of 72°C for 5 minutes.

PCR products were purified by digestion with 0.5 U shrimp alkaline phosphatase (rSAP; New England Biolabs, Massachusetts, USA) and 2.5 U exonuclease I (Exo I; New England Biolabs, Massachusetts, USA) at 37°C for 15 min, followed by inactivation of the enzymes by 15 min at 80°C. DNA sequencing was performed by capillary separation at the Massey Genome Service (Palmerston North, New Zealand) or Macrogen (Seoul, South Korea).

Sequences were edited in Sequencer 5.4.6 (Gene Codes Corporation). Sequences were aligned to publicly available fairy and fulmar prion sequences (S1 Table) by eye (no insertion/deletion events (indels) were present in either COI or cytochrome *b*).

### mtDNA sequencing analysis

To test whether the COI sequences deviated from neutral expectations we performed Ewans-Watterson and Chakraborty tests [34–36] in ARLEQUIN 3.5.1.2 [37].

Estimates of population differentiation between colonies (G_st_, N_st_ and Φ_st_) at the COI locus were calculated using SPADS v1.0 [38]. Statistical significance of these values was assessed by 10 000 random permutations. G_st_ is calculated using only haplotype frequency data, whereas N_st_ takes the relationships between haplotypes into account and thus provides an indication of the extent of phylogeographical signal. G_st_ and N_st_ were compared using a permutation test with 10 000 permutations. If N_st_ is significantly greater than G_st_, it suggests that haplotypes in close geographic proximity are more likely to also have a close genetic relationship [39].

The geographic structure of the COI variation in fairy and fulmar prions was examined by spatial analysis of molecular variance (SAMOVA [40]), implemented in SPADS 1.0. SAMOVA defines groups of populations by maximizing the proportion of the total genetic variance due to differences between groups of populations (F_CT_). The number of groups (*K*) was set to vary between 2 and 8, with SAMOVA run with 10 000 iterations and 10 repetitions. The optimal *K* was selected by choosing the highest F_CT_ where no groups comprised of samples from a single site.

The partitioning of COI molecular variance within prions was tested with analyses of molecular variance (AMOVA) using ARLEQUIN 3.5.1.2. Significance was tested through 10 000 permutations. Populations were grouped either by species, geography or the optimal grouping (*K*) determined by SAMOVA. For the geography grouping, prions within mainland New Zealand were grouped into either a North Island (Poor Knights Islands) or South Island (Mana Island, which were themselves translocated from Stephens Island/Takapourewa in the South Island, Otago, West Coast and NE Stewart Island) group. Under the geography grouping, birds from the same island archipelago were also grouped (i.e. fairy and fulmar prions from the Chatham Islands, and fairy and fulmar prions from the Snares Islands).

Phylogenetic analyses were performed using maximum likelihood (ML) and Bayesian Inference (BI). COI and cytochrome *b* sequences were analysed both independently and with the two loci combined for only the samples with both loci sequenced.

ML analyses were performed with the PhyML v3.0 web server (http://www.atgc-montpellier.fr/phyml/ [41]), with the best-fit model of sequence evolution determined with Smart Model Selection (HKY85 + G for both loci plus the combined dataset) and the Akaike information criterion [42]. Heuristic searches were performed with 10 random addition sequence replicates and SPR branch-swapping. Branch support was assessed with 1000 bootstrap (BS) pseudoreplicates.

MrBayes v3.2.1 [43] was used to perform BI. Two concurrent analyses were run, each with four Markov chains of fifty million generations and sampling every 1000 generations, nst = 6, rates = invgamma and default priors. The first 20% of samples were discarded as ‘‘burn-in”, after this point the standard deviation of split frequencies was below 0.01 and Tracer v.1.71 [44] also confirmed that stationarity had been reached.

Genealogical relationships between mtDNA sequences were determined using median joining networks [45] produced with PopART [46]. The loci were analysed separately because neither locus was available for all locations e.g., only cytochrome *b* sequences were available for the Falkland Island birds.

### ddRAD sequencing

Double-digest restriction site-associated DNA sequencing (ddRADseq) libraries [24] were prepared for 21 fairy prions, 17 fulmar prions and six outgroups (S3 Table). Four samples were processed in duplicate as technical replicates, including one sample that was included on both sequencing runs. Libraries were prepared following the method of [47] and described at [48].

Briefly, for each sample, 250 ng of DNA was digested with two restriction enzymes, following manufacturer’s instructions. We tested AvaII (New England Biolabs, Massachusetts, USA) paired with MspI or MseI (New England Biolabs, Massachusetts, USA). AvaII and MspI were selected because they produced a large number of fragments within the desired size range for the test sample. Adaptors containing sample-specific barcodes and Illumina indices were ligated to each sample. Samples were pooled into three index pools and 300–500 bp fragments were size-selected by excision from an agarose gel, followed by extraction with a Qiaquick gel extraction kit (Qiagen). Illumina indices were then added by PCR to each size-selected sample pool with Phusion flash high fidelity PCR master mix (Thermo Scientific). Each pooled sample was purified and concentrated with a MinElute kit (Qiagen), quantified with a Qubit dsDNA high sensitivity assay kit (Thermo Fisher Scientific) and combined in equimolar amounts. The library was sequenced across a quarter of a lane of an Illumina HiSeq 2500 to generate 2 × 125 bp reads. A second library was prepared, as described above, from either re-extractions of samples that failed on the first run or from samples acquired after the first run was performed. For the second run, samples were combined into a single index pool and sequenced across an eighth of a lane, as described above.

### ddRAD sequencing analysis

For the samples with sufficient numbers of reads (see Results) the paired-end reads were demultiplexed, had their adaptors removed, quality filtered, merged and assembled into *de novo* loci with ipyrad v0.7.28 [49]. Reads were clustered at 85% similarity, with clusters with a depth of coverage fewer than six and more than 10 000 excluded. A maximum of two nucleotides per site were permitted and consensus sequences with more than 50% heterozygous sites were excluded in order to reduce the inclusion of paralogous regions. Loci present in at least 26 of the 36 samples were retained. Following the exclusion of samples with a low number of assembled loci (see Results), the reads of the remaining samples were re-clustered, as described above, and duplicate samples combined to create a final dataset retaining loci present in at least 24 of the remaining 25 samples.

A network of the ddRADseq data, with constant sites omitted, was constructed with the NeighborNet algorithm [50], implemented in SplitsTree4 v4.14.8 [51] using uncorrected p-distances and the equal angle algorithm. Support was assessed with 1000 bootstrap replicates. A phylogenetic tree was constructed from the ddRADseq data, with constant sites omitted, using ML analysis with IQ-TREE [52] on the IQ-TREE web server [53]. The best model of nucleotide substitution and across-site heterogeneity in evolutionary rates (GTR+F+ASC+R2) was inferred using ModelFinder [54], based on the corrected Akaike’s information criterion. Support was assessed with 1000 ultrafast bootstraps (UF-BS) [55] and the Shimodaira-Hasegawa approximate likelihood ratio test (SH-aLRT [41]) with 1000 replicates. A second phylogeny was constructed as described above using a single unlinked biallelic SNP extracted from each locus with vcftools [56].

We performed topological tests in IQ-TREE to assess the robustness of the reconstructed ML phylogeny containing all SNPs compared to a phylogeny where fairy prions and fulmar prions were each constrained to be monophyletic, i.e., (outgroups (fairy prions)(fulmar prions)). The likelihoods of the two trees were compared using a Kishino-Hasegawa (KH) test [57], a Shimodaira-Hasegawa (SH) test [58] and an expected likelihood weight (ELW) test [59].

The *D* statistic [60], or ABBA-BABA test, is a test that analyses allelic patterns in order to distinguish between hybridisation and incomplete lineage sorting. The *D* statistic is calculated from the number of allele distribution patterns that conflict with the four-taxon tree (outgroup, (P3,(P2,P1))). The “ABBA” and “BABA” patterns should occur equally (*D* = 0) if incomplete lineage sorting is the only process occurring. In contrast, gene flow between P3 and P1 or P2 will result in an excess of either “ABBA” or “BABA” patterns. We ran all possible four-taxon *D* statistics with *Dtrios* in the DSUITE software [61]. Tests were performed on a dataset of 9759 biallelic SNPs, compiled by extracting a single SNP from each locus using vcftools [56]. The groups tested were Snares Islands fairy prions, Snares Islands fulmar prions, Chatham Islands fairy prions, Chatham Islands fulmar prions and Auckland Islands fulmar prions. Significance was assessed using jackknife [62] on blocks of 20 SNPs. To further test for signals of hybridisation and introgression we performed HyDe analyses [63]. HyDe detects hybridisation using phylogenetic invariants based on the coalescent model with hybridisation and provides an estimate of the amount of admixture (γ). We ran HyDe with individuals pooled into the same groups as were analysed for *D* statistics. We ran a second analysis with no pooling of individuals (i.e. each individual sample considered a separate entity).

Population structuring of the fairy and fulmar prion ddRADseq data was examined with STRUCTURE v2.3.4 [64] on the EasyParallel platform [65] and with sNMF, which uses sparse nonnegative matrix factorisation and least-squares optimisation [66]. For STRUCTURE a single SNP was randomly selected from each ddRAD locus (.ustr file) using ipyrad v0.7.28. The number of genetic clusters (*K*) was set between 1 and 5, with 3 permutations performed for each. We used the admixture model with correlated allele frequencies and ran STRUCTURE with a burn-in of 50,000 generations, followed by 100,000 Markov Chain Monte Carlo (MCMC) iterations. A longer run was also performed once for *K* = 3 for the dataset which included the outgroups (100,000 generation burn-in and 500,000 MCMC iterations). The optimal number of genetic clusters (*K*) was determined by calculating the *ΔK* statistic [67] in STRUCTURE HARVESTER web v.0.6.94 [68]; however, we also examined all clustering results that warranted biological interpretation, following [69]. The CLUMPAK online server (http://clumpak.tau.ac.il/contact.html [70]) was used to average iterative runs of *K* and visualise the results.

For sNMF, which has the advantage over STRUCTURE of avoiding Hardy-Weinberg equilibrium assumptions [66], ancestry co-efficients were calculated for *K* of 1 to 5, with 5 replicates for each *K*. Tests were performed on a.vcf file containing only a single unlinked biallelic SNP from each locus extracted with vcftools [56]. The optimal *K*-value was selected using a cross-entropy criterion based on the prediction of masked genotypes to evaluate the error of ancestry estimation.

We also explored ddRADseq variation among the samples by performing a principal component analysis (PCA) using the ipyrad-analysis toolkit (https://ipyrad.readthedocs.io/en/latest/API-analysis/cookbook-pca.html [49]. Twenty-five replicate analyses were run, each subsampling a different random set of unlinked SNPs. Kmeans clustering was performed with *K* = 5 in order to impute missing values in the dataset.

## Results

### mtDNA sequences

DNA could not be amplified from either of the Holocene-aged fossil bones (S2 Table). The oldest study skins that were successfully amplified were collected in 1950 (Fairy18-Fairy20; S1 Table).

None of the Ewens-Watterson’s and Chakraborty’s tests were significant (all p > 0.05), indicating no departure from neutrality for the mtDNA sequences. Translation of the cytochrome *b* and COI sequences indicated that there were no internal stop codons.

The COI and cytochrome *b* alignments were 774 bp and 834 bp in length, respectively. We found 31 haplotypes among the 92 fairy and fulmar prion COI sequences, defined by 30 variable sites. For cytochrome *b* we found 23 haplotypes, defined by 56 variable sites, from the 42 fairy and fulmar prion sequences. For the combined COI and cytochrome *b* dataset of 42 fairy and fulmar prion sequences, the alignment length was 1608 bp, distinguishing 30 haplotypes with 46 variable sites.

There were no fixed mutations differentiating fulmar and fairy prions at COI or cytochrome *b*. Private COI haplotypes were found in all colonies except Bass Strait, Kerguelen Islands, Herekopare Island, and the Auckland Islands, although few birds were sequenced from these colonies. Of the 31 COI haplotypes detected only five were found in more than one colony. Cytochrome *b* showed a similar pattern.

**Fig 2:**
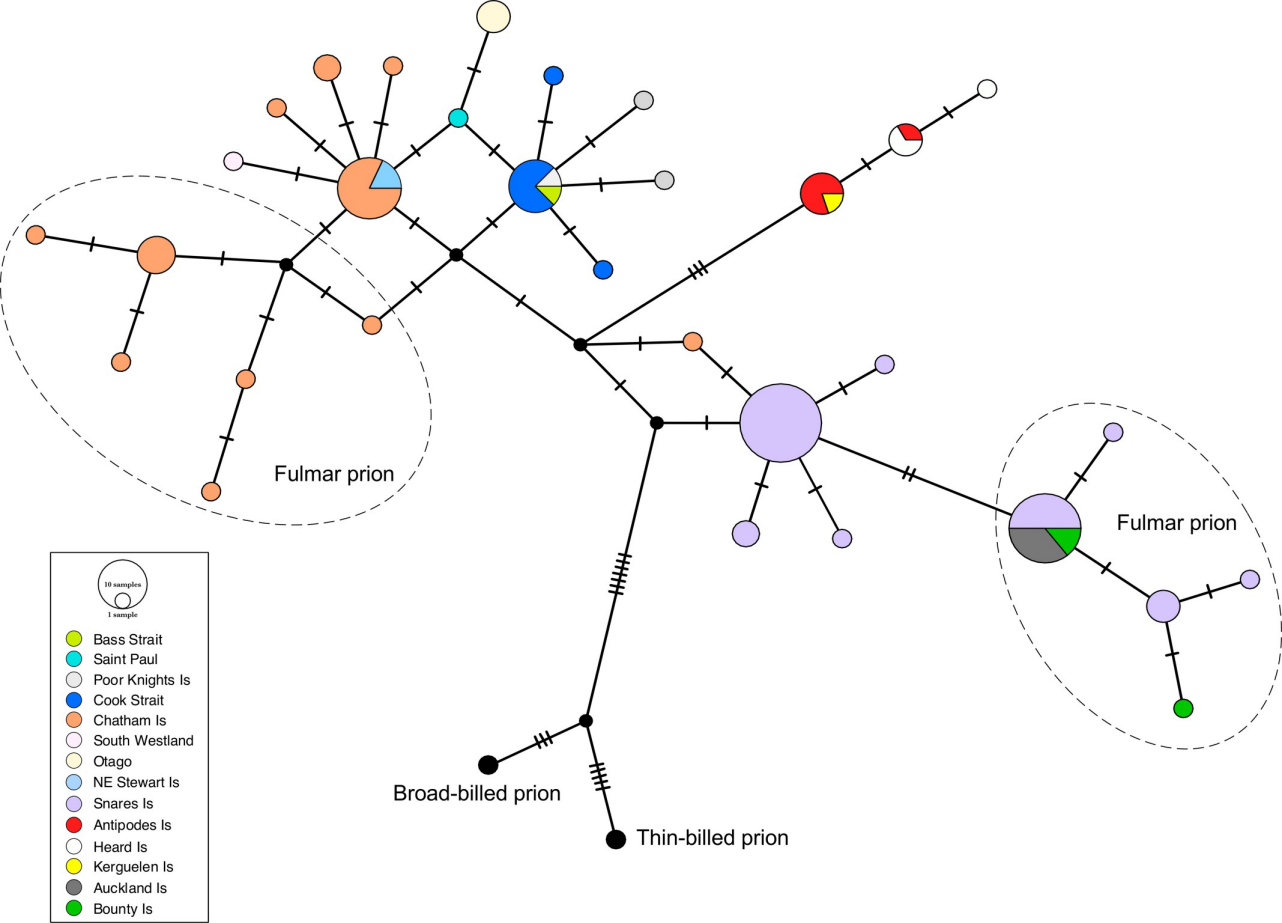
COI median joining network of fairy and fulmar prions with colour-coded sampling localities. Fulmar prion samples are encompassed by a dashed line. The size of each circle is proportional to haplotype frequency. Hatch marks represent additional mutational steps separating haplotypes.

**Fig 3:**
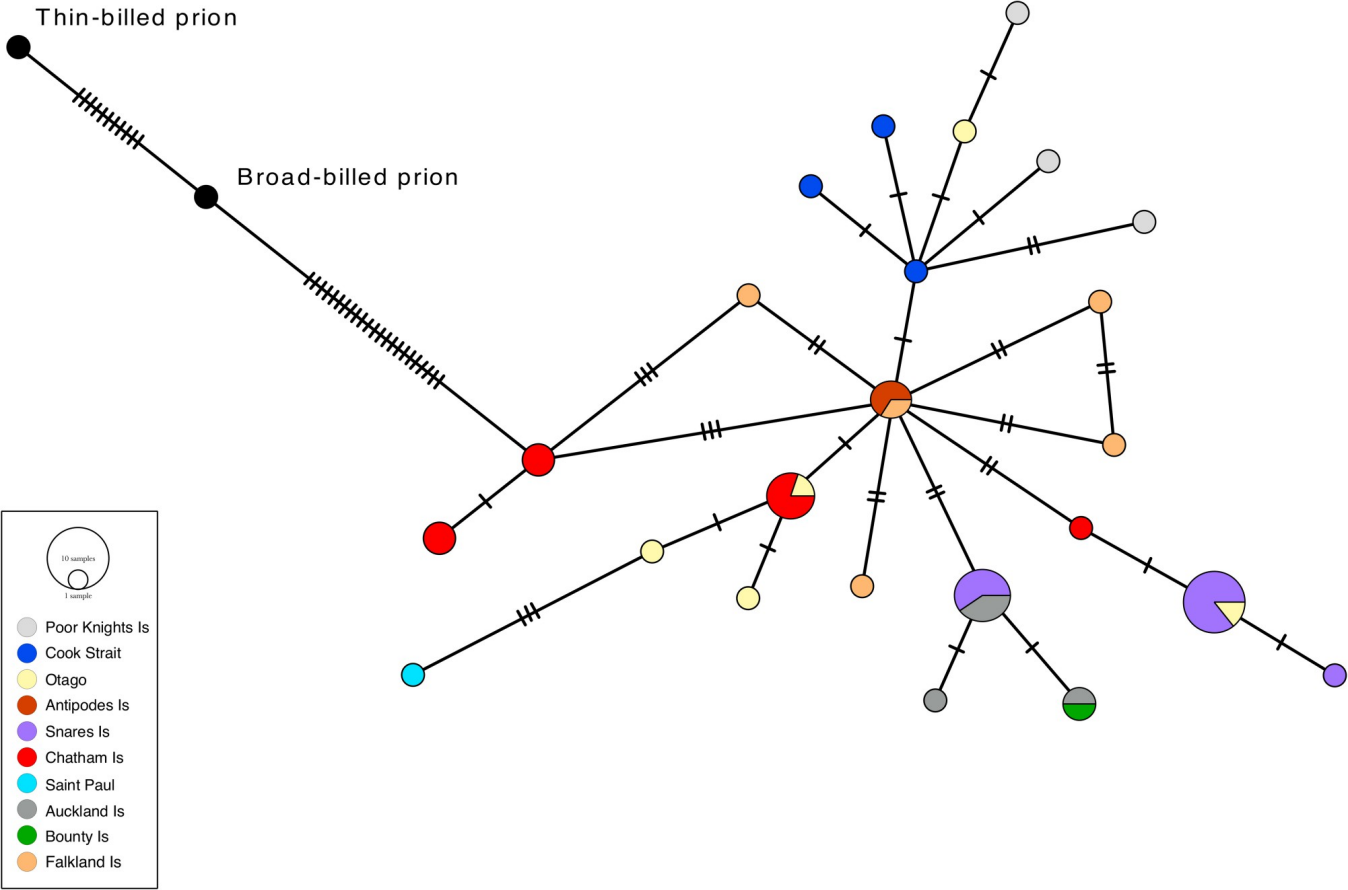
Cytochrome *b* median joining network of fairy and fulmar prions with colour-coded sampling localities. Fulmar prion samples are encompassed by a dashed line. The size of each circle is proportional to haplotype frequency. Hatch marks represent additional mutational steps separating haplotypes.

The relationships among the COI haplotypes are shown in the median-joining network (Fig 2). The maximum number of mutational steps between the ingroup COI haplotypes was 11. The outgroups joined the COI network at an intermediate haplotype closest to a fairy prion haplotype from the Snares Islands. The COI haplotypes generally clustered geographically. However, a Chatham Island fairy prion haplotype was more closely related to a Snares Islands fairy prion haplotype than to the other Chatham Island haplotypes (Fig 2). The cytochrome *b* network (Fig 3) was similar in topology to the COI network but showed less resolution and the outgroups connected to a Chatham Island fulmar prion sequence.

Population differentiation between colonies was moderate to high (G_st_ = 0.378; N_st_ = 0.827). N_st_ was significantly higher than G_st_ (P < 0.0001) suggesting there was a phylogeographic component to the structuring. The global Φ_st_ was also high (0.817; P < 0.0001).

For the SAMOVA analysis the highest F_CT_ value with no groupings of single populations was *K* = 3 (F_CT_ = 0.581). At *K* = 3, group 1 consisted of fairy prions from the Snares and fulmar prions from the Snares, Bounty, and Auckland Islands. Group 2 contained fairy prions from the Antipodes, Heard, and Kerguelen Islands and Group 3 contained the remaining populations. These groupings are in concordance with both the COI and cytochrome *b* haplotype networks.

The AMOVA of the COI sequences indicated that geography and SAMOVA group explained much more of the haplotype partitioning (41.61% and 49.51%, respectively) than species boundaries (0.56%); however, for both analyses nearly half of the total haplotype variation was attributed to variation within populations (S4 Table). The F_CT_ value was not significant when populations were partitioned by species, which likely results from the low power associated with analysing only two species [71].

**Fig 4:**
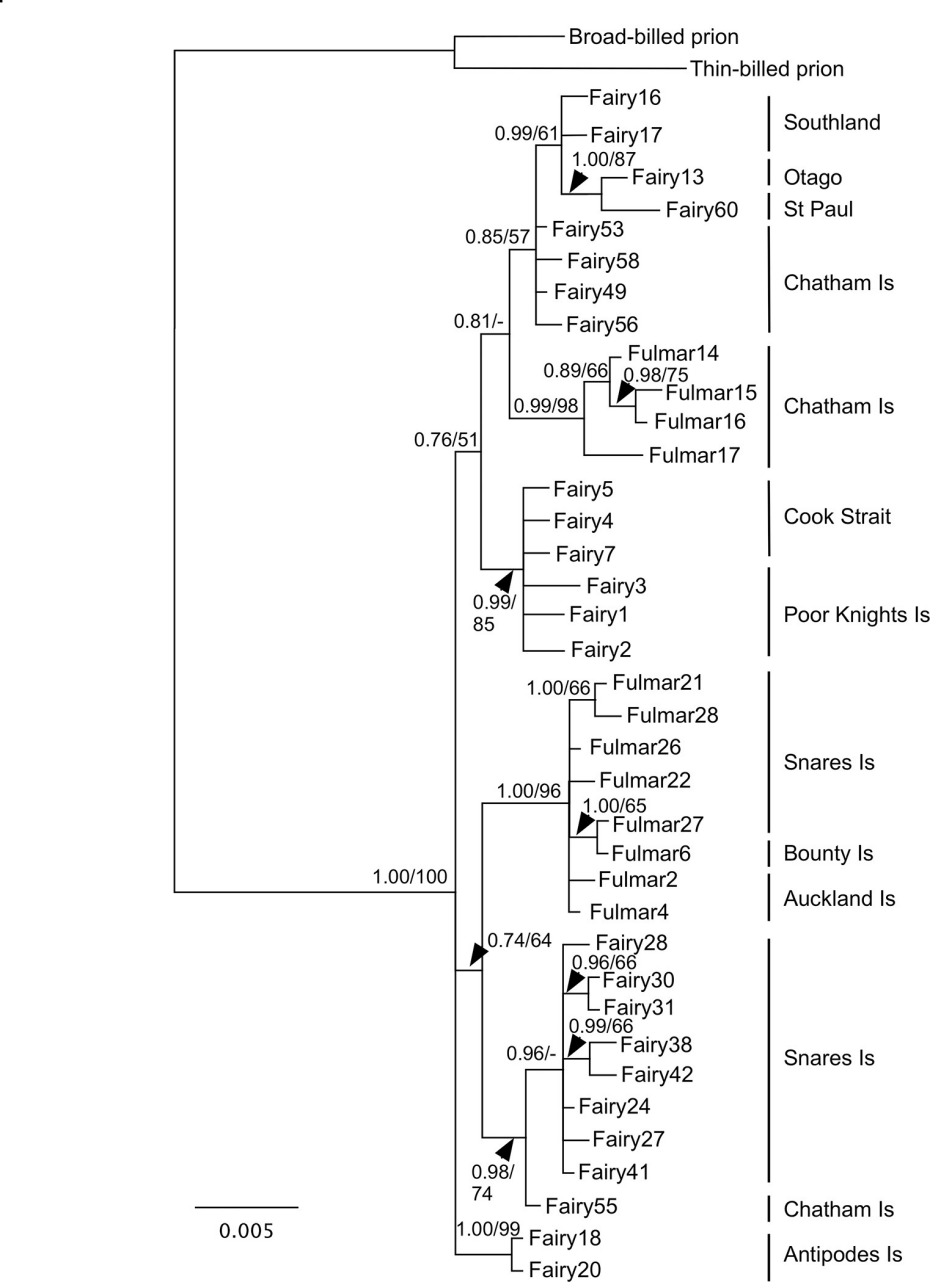
Bayesian phylogeny of concatenated COI and cytochrome *b* sequences for fairy and fulmar prions. Support values for nodes are as follows: Bayesian posterior probability/maximum likelihood bootstrap.

The topologies of the mtDNA phylogenies showed little conflict, so only the concatenated COI and cytochrome *b* Bayesian phylogeny, which had the best resolution, is shown (Fig 4). Many of the shallow relationships in the phylogeny are well-supported but the deeper relationships are not. The fulmar prion sequences from the Chatham Islands formed a well-supported clade (0.99 PP/98% ML BS), as did the fulmar prions from the Snares Islands, Auckland Islands and Bounty Islands (1.00 PP/96% ML BS). These two fulmar prions lineages were more closely related to fairy prions than to each other; however, support for these relationships was weak.

### ddRADseq

The Illumina sequencing of the 44 prion ddRADseq libraries resulted in 147 million (M) raw paired reads. Eight samples were initially excluded owing to low numbers of reads (S3 Table). Following clustering with an 85% similarity threshold, a further nine samples had low numbers of loci and were also excluded from the final dataset. Of the four samples prepared in duplicate, two had low numbers of assembled loci and were excluded. The other two replicates grouped with their duplicates in a preliminary NeighborNet network (S1 Fig). The final dataset, with low coverage samples removed and duplicates combined, had a total of 117M paired reads, with an average of 4.7M paired reads per individual. Across the 25 individuals, this final assembly comprised 9791 loci with 30677 SNPs.

**Fig 5:**
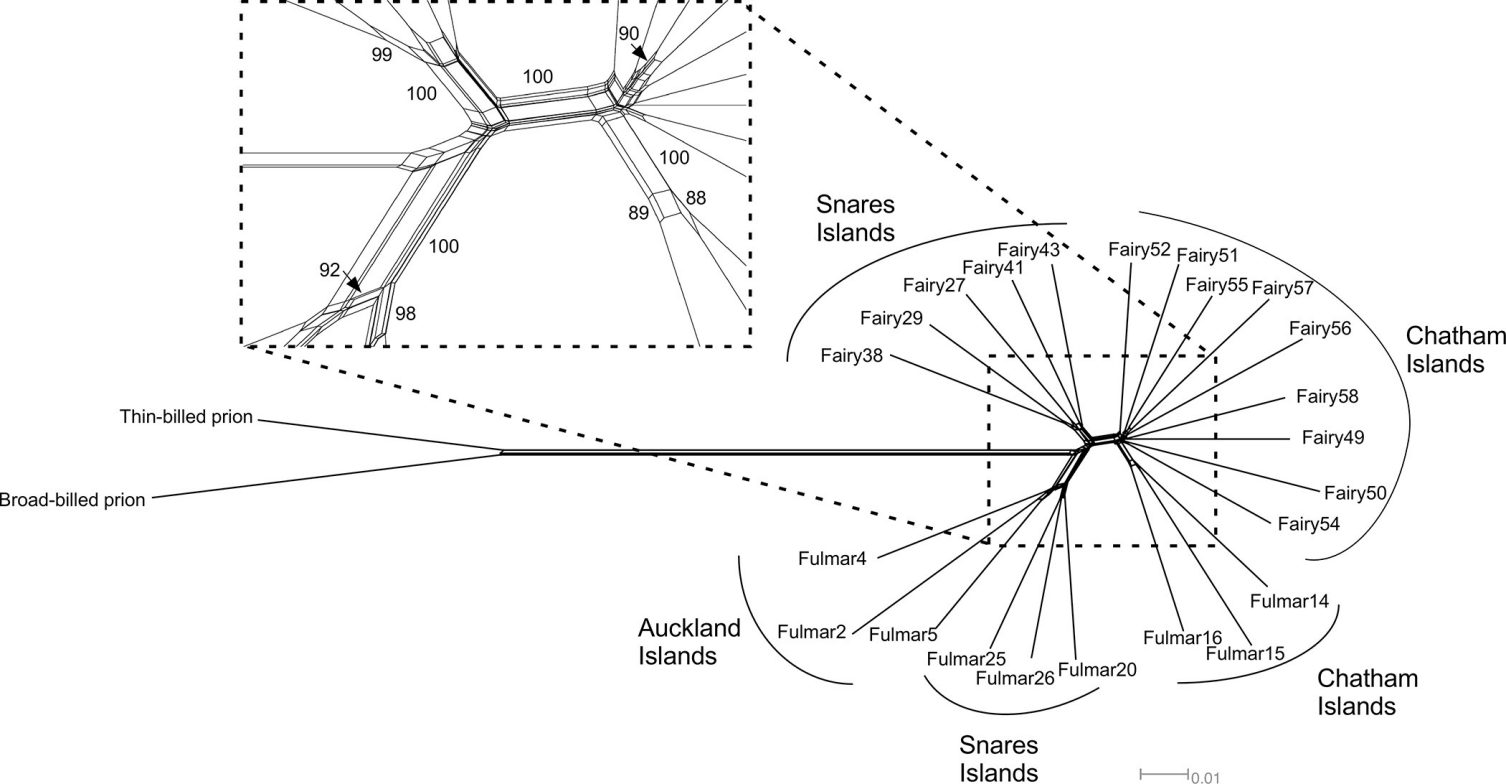
NeighborNet phylogenetic network derived from 30677 SNPs from 9791 ddRADseq loci. Bootstrap support values over 80% are shown.

In the NeighborNet network (Fig 5) clustering occurred by geography rather than by species. Fulmar prions from the Chatham Islands were recovered as a well-supported cluster (100% BS), as were fairy prions from the Snares Islands (100% BS) and fulmar prions from the Snares Islands (98% BS). There was little support for the fairy prions from the Chatham Islands forming a cluster but they did group with fulmar prions from the Chatham Islands (100% BS). A cluster containing fulmar prions from the Snares and Auckland Islands also received strong support (100% BS).

**Fig 6:**
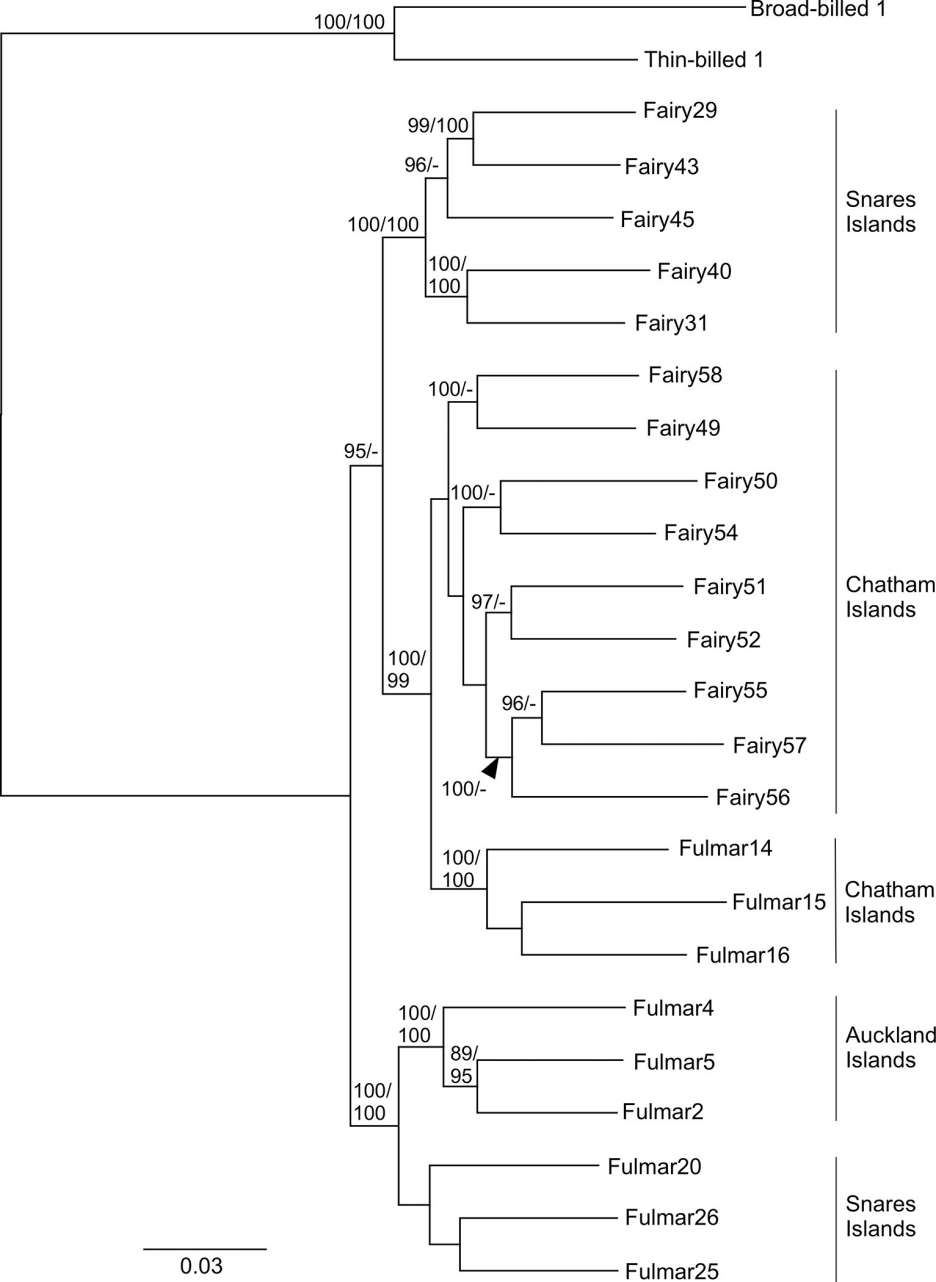
Maximum likelihood phylogeny constructed from 30677 SNPs from 9791 ddRADseq loci. Branch support values are given in the order SH-aLRT/UF-BS and are only shown when they are ≥ 80%for SH-aLRT and ≥ 95% for UF-BS.

The maximum likelihood phylogeny (Fig 6) constructed from all the SNPs in IQ-tree had a similar topology to the network. Three main clades were recovered, each with 99–100% SH-aLRT and 100% ultrafast bootstrap support (UF-BS): Snares fairy prions, Auckland Island plus Snares Island fulmar prions, and Chatham Island fairy plus fulmar prions. However, the relationships between these clades were not well-supported (79% SH-aLRT/93% UF-BS). The clade of Chatham Island fulmar prions was well-supported (100% SH-aLRT /100% UF-BS) and was recovered as sister to Chatham Island fairy prions (100% SH-aLRT /99% UF-BS). The phylogeny constructed from a single SNP per locus (S2 Fig) had much lower support values, as expected for this reduced dataset. The main difference to the phylogeny constructed with all the SNPs was that Snares Island fairy prions were recovered in a well-supported clade with the Snares Island plus Auckland Island fulmar prions (99% SH-aLRT/97% UF-BS) in the single SNP per locus phylogeny.

The topology that constrained the groupings by species (outgroups (fairy prions)(fulmar prions)) was rejected by the KH and SH tests (p < 0.0001). The c-ELW test also rejected this topology, providing a posterior weight of 1 to the alternative topology of (outgroups (Chathams fairy and fulmars) (Snares fairy and fulmars, Auckland Islands fulmars)).

The 10 pairwise *D* statistics calculated between Snares Islands fairy prions, Snares Islands fulmar prions, Chatham Islands fairy prions, Chatham Islands fulmar prions and Auckland Islands fulmar prions ranged from 0.002 to 0.018. None was significant (p > 0.2), indicating that there is no signal of hybridisation. Furthermore, no hybridisation events were inferred from the HyDe analyses.

**Fig 7:**
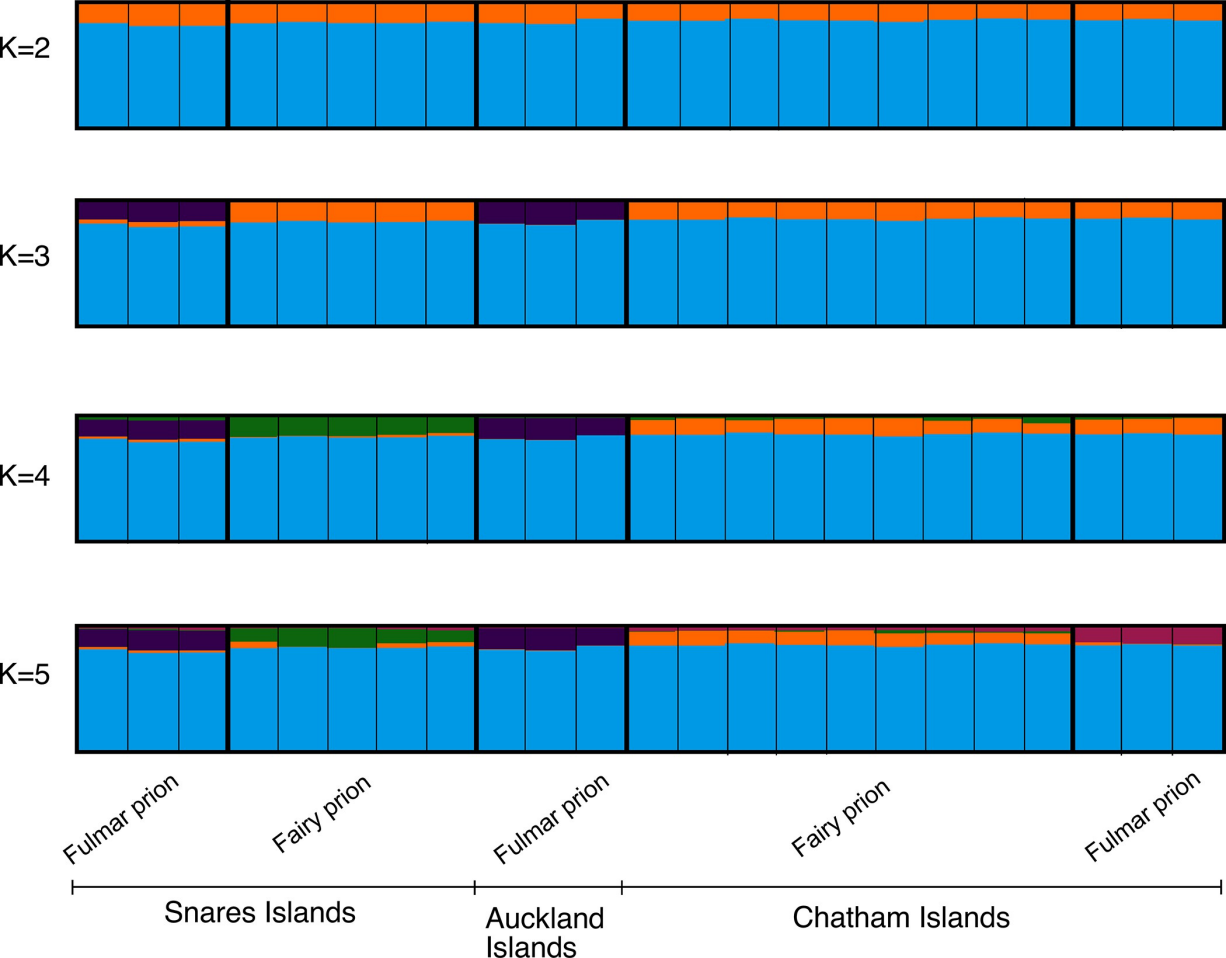
STRUCTURE plot of fairy and fulmar prions for *K* = 2 to *K* = 5 based on 9791 ddRADseq SNPs.

The Structure Harvester analysis of Δ*K* indicated that the optimal *K* was 2, although Δ*K* does not assess *K* = 1 as a possible solution. At *K* = 2 all fairy and fulmar prions were partitioned into the same cluster with high probability (Fig 7). At values of *K*>2 fairy and fulmar prions were still grouped into a single cluster with high probability but each individual was also estimated to have a minor proportion of their Q-value assigned to an additional cluster (Fig 7). These minor groups partitioned off fulmar prions from the Snares Islands and Auckland Islands at *K* = 3, fairy prions from the Snares Islands at *K* = 4 and fulmar prions from the Chatham Islands at *K* = 5 (Fig 7).

**Fig 8:**
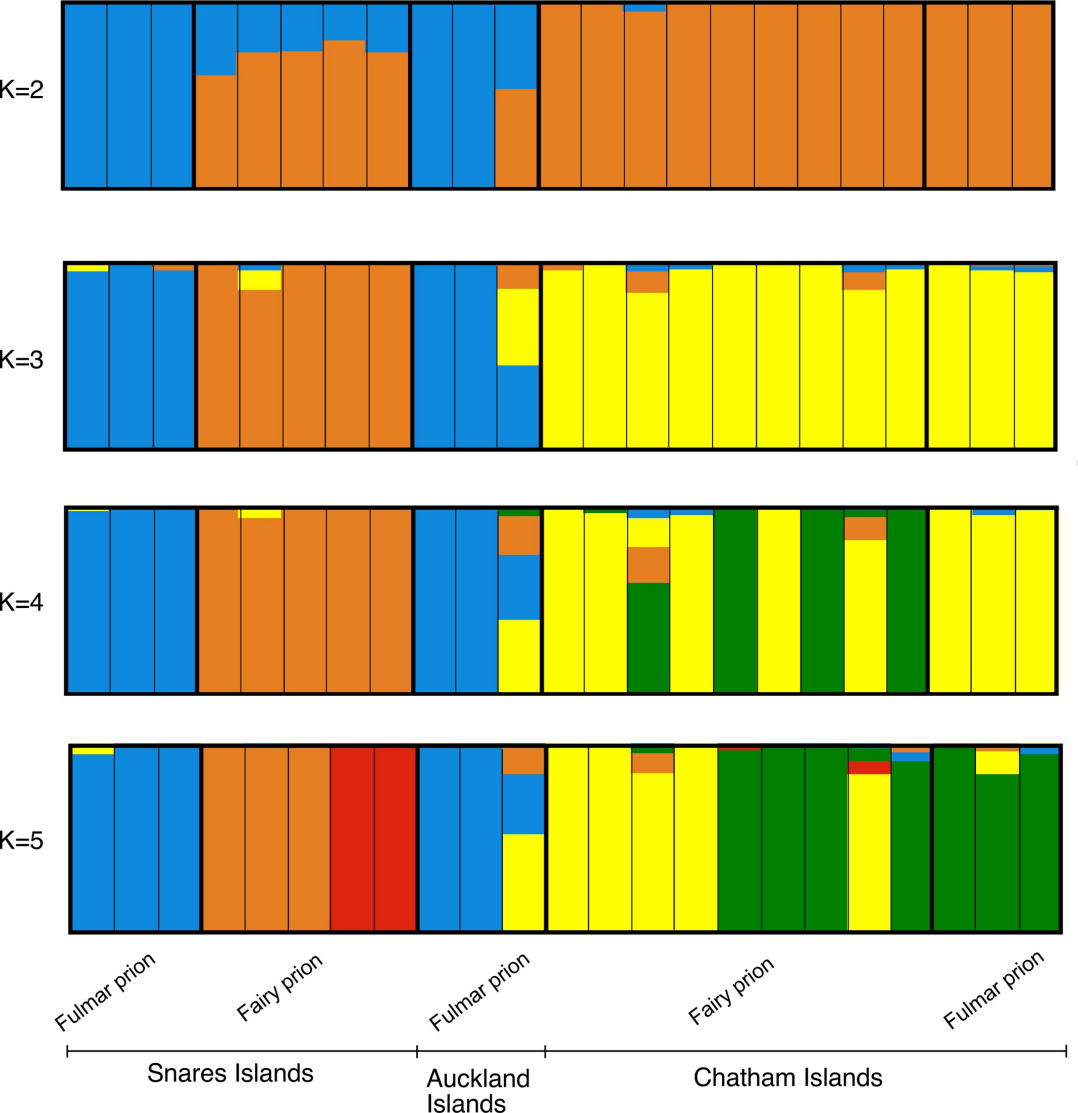
Population structuring determined by sNMF analysis for fairy and fulmar prions ddRADseq SNPs for *K* = 2 to *K* = 5.

The sNMF analysis (Fig 8) revealed more pronounced genetic structuring patterns than the STRUCTURE analysis. *K* = 1 had the lowest cross-entropy value, indicating this was the optimal *K*. However, other values of *K* produced clustering results that also warrant biological interpretation [69]. At *K* = 2 fulmar prions from the Snares and Auckland Islands were partitioned from the remaining samples. At *K* = 3 an additional cluster comprising fairy and fulmar prions from the Chatham Islands was partitioned. At *K* = 4 partitioning within Chatham Islands fairy prions was evident and at *K* = 5 Snares Islands fairy prions were separated into two clusters. Unlike the STRUCTURE results fulmar prions from the Chatham Islands were not distinguished as a separate cluster.

In a preliminary PCA sample Fulmar 2 from the Auckland Islands was an outlier, likely owing to its high proportion of missing data (S3 Table), so was subsequently excluded. In the final PCA Snares Islands fairy prions formed a distinct cluster that was separated from other samples along the PC0 and PC1 axes (S3 Fig). A Chatham Islands fairy prion cluster and Chatham Islands fulmar prion cluster were evident with each separated from the remaining samples along the PC2 axis (S4 Fig). The Snares Island fulmar prions and Auckland Island prions were not distinguishable.

## Discussion

### Phylogeny does not reflect morphology

Neither fairy prions nor fulmar prions were recovered as monophyletic in analyses of the mtDNA and ddRADseq data. Furthermore, an alternative topology enforcing monophyly of each of these groups was rejected by topology tests of the ddRADseq data. Instead, our data showed geographical partitioning of genetic variation across fairy and fulmar prions, which was particularly pronounced in the mtDNA data, indicating that there has been parallel phenotypic evolution of bill morphology within the fairy/fulmar prion clade. The *D* statistics of the nuclear data suggested a lack of gene flow between lineages, indicating that the observed phylogenetic relationships are not a result of hybridisation. Furthermore, the morphological and genetic distinctiveness of prion lineages occurring in near sympatry, with no obvious physical barriers to gene flow, at the Snares Islands (7 km minimum distance between known colonies of fairy and fulmar prions) and Chatham Islands (25 km minimum distance) demonstrates that each is a distinct evolutionary lineage and should therefore be recognised at species rank (see Taxonomy section).

### Speciation within the fairy/fulmar prion complex

Friesen [26] listed six factors that potentially have a role in seabird speciation: physical isolation, breeding phenology (allochrony), differences in ocean regime, philopatry, non-breeding distributions and foraging distributions during the breeding season. Given the lack of either contemporary or historical physical barriers in the Southern Ocean [27] and the close proximity of some fairy and fulmar prion colonies, physical isolation is unlikely to have contributed to the observed genetic patterns.

Allochrony (differences in breeding time) has been reported between other sympatric prion species on the Crozet Islands [7], Kerguelen Islands [72] and Gough Island [73]. However, fairy and fulmar prions breed at a similar time of year [16].

Differences in ocean regime may have resulted in restricted gene flow between distant fairy and fulmar prion colonies but would not have produced the genetically distinct lineages in close proximity within the Snares or Chatham Island archipelagos. Philopatry has been reported in fairy prions (see Introduction) indicating that it may have had a role in restricting gene flow between colonies and producing the observed genetic structuring. However, philopatry usually acts in combination with other barriers to gene flow [26].

The non-breeding distributions and foraging distributions during the breeding season, which can reduce competition and opportunities for gene flow, are unknown in fairy and fulmar prions. Other sympatric prion species have been reported to segregate during the nonbreeding period [74, 75] but non-breeding distributions were not linked to genetic structure in Antarctic and thin-billed prions [76]. The sympatric fairy and fulmar prion lineages have different foraging strategies. Fairy prions mainly eat small pelagic crustaceans, along with small fish and squid [17]. Chatham Island fulmar prions mostly eat adult barnacles, while Snares Islands fulmar prions largely feed on crustaceans (euphausiids and amphipods) [77]. This niche partitioning may have evolved as a mechanism to reduce competition between adjacent colonies, particularly during the breeding season, and has also been suggested to explain the high genetic divergence detected between adjacent colonies of Hawaiian petrel [78].

Breeding habitat characteristics may also have contributed to genetic differentiation between prion lineages by influencing choice of breeding site. Fulmar prions usually nest in natural crevices, rock piles and caves [16, 77], whereas fairy prions mainly breed in burrows [17]. Birds may be reluctant to shift between these different habitats. Another behavioural difference between fulmar and fairy prions is that fulmar prions have a tendency to be diurnal at many breeding sites, whereas fairy prions are invariably nocturnal [17, 77].

### Post-LGM colonisation

The distribution of and relationships between lineages in the fairy/fulmar prion complex is similar to many Southern Ocean taxa and is consistent with colonisation from refugia following the Last Glacial Maximum (LGM). Our COI network provides evidence of postglacial dispersal from islands known to be refugia during the LGM to islands that were glaciated, a pattern also observed in other Procellariiformes [79], as well as unrelated taxa such as several penguin species [80] southern bull-kelp [81]. For example, fairy prions from Kerguelen and Heard, which were glaciated during the LGM, appear to derive from prions from the Antipodes Islands, a known LGM refugium ([27] and references therein). Similarly, the Auckland Islands were moderately glaciated during the LGM [27] and their fulmar prions appear to have colonised from the unglaciated Snares Islands.

### Taxonomy

Our mtDNA and nuclear sequences revealed multiple genetically and morphologically distinct lineages within the fairy/fulmar prion complex. These results are incongruent with the current taxonomy because fulmar prions are not monophyletic in our mtDNA and nuclear phylogenies. One option to resolve this conundrum would be to consider fairy and fulmar prions a single widespread, but morphologically variable, species (*sensu* [5, 9]). However, the co-occurrence of lineages that are morphologically and genetically distinct in near sympatry at two locations (Chathams and Snares Islands) indicates the presence of multiple non-interbreeding species.

Within fairy prions, we recovered a number of genetically distinct clades. The fairy prions from Kerguelen Island, Heard Island and the Antipodes Islands formed a distinct lineage in the COI network, but they were not able to be included in our ddRADseq dataset owing to poor-quality DNA from museum specimens (all are remote breeding sites, making obtaining blood samples from these locations challenging). We recognise these birds as a subspecies of fairy prion. Notably we conclude that the Heard Island birds are fairy prions, rather than fulmar prions, which resolves the uncertainty around the relationship of these birds (see [22]). We retain a single species (and subspecies) encompassing the remaining diversity within fairy prions until more data (morphological and nuclear DNA) can be obtained.

### Taxonomic recommendation

Order Procellariiformes Fürbringer, 1888

Family Procellariidae Leach, 1820

Genus *Pachyptila* Illiger, 1811

### Fairy prion *Pachyptila turtur turtur* (Kuhl, 1820)

Type locality = Bass Strait, Australia (fide [82]).

Breeds on many islands in the New Zealand region including the Poor Knights Islands, Cook Strait islands, West Coast islands, Banks Peninsula islands, Otago islands, Foveaux Strait and Stewart Island islands, Snares Islands, Chatham Islands (Mangere, Little Mangere, Rabbit, Kokope, Murumurus, Star Keys, The Sisters) and on the mainland at Dunedin, in Australia on islands in Bass Strait, and St Paul Island, Indian Ocean [16, 19]

### Subantarctic fairy prion *Pachyptila turtur eatoni* (Mathews, 1912)

Type locality: Kerguelen Island.

Breeds on Kerguelen Islands, Heard Island, and the Antipodes Islands; presumed to also breed on the Falkland Islands, South Georgia, Crozet, Prince Edward, and Macquarie Islands, and Bishop Islet [16, 83].

### The Pyramid prion *Pachyptila pyramidalis* C.A. Fleming, 1939

Type locality: The Pyramid, Chatham Islands.

Breeds on The Pyramid and The Forty Fours, Chatham Islands [19].

### Fulmar prion *Pachyptila crassirostris crassirostris* (Mathews, 1912)

Type locality: Bounty Islands.

Breeds on the Bounty Islands, and Toru and Rima islets of the Western Chain, Snares Islands [18, 20].

### Lesser fulmar prion *Pachyptila crassirostris flemingi* Tennyson & Bartle, 2005

Type locality: Ewing Island, Auckland Islands.

Breeds on Ewing, Ocean, Rose, Disappointment, and Monumental Islands, Auckland Islands [20].

### Conservation implications

*Pachyptila pyramidalis* (formerly *P*. *crassirostris pyramidalis*), *P*. *crassirostris flemingi* and *P*. *crassirostris crassirostris* are all endemic to New Zealand. *Pachyptila pyramidalis* and *P*. *c*. *crassirostris* are currently classified as ‘At Risk–Naturally Uncommon’ under the New Zealand Threat Classification System [83]. *Pachyptila c*. *flemingi* is classified as Threatened–Nationally Vulnerable because its small population is likely a consequence of the impacts of introduced mammals on islands within the Auckland Islands [21, 84, 85].

*Pachyptila turtur* was considered to be a Relict taxon under the New Zealand Threat Classification System [84]. *Pachyptila turtur turtur* also meets the criteria for this classification. Tens of thousands of pairs of *P*. *turtur eatoni* occur on some islands, e.g. Kerguelen and the Crozet Islands [16]. However, only between 1000 and 5000 pairs have been recorded from the only New Zealand population, which occurs on the Antipodes Islands [86]. Under the New Zealand Threat Classification System [87], *P*. *turtur eatoni* should be classified as ‘Naturally Uncommon’, with the qualifiers ‘Secure Overseas’ and ‘One Location’.

## Supporting information

S1 Fig Preliminary NeighborNet phylogenetic network derived from ddRADseq loci. Bootstrap support values over 80% are shown. Duplicates are indicated by ‘dup’ after sequence name and were combined for subsequent analyses.
(PDF)

S2 Fig Maximum likelihood phylogeny constructed from 9791 unlinked SNPs. Branch support values are given in the order SH-aLRT/UF-BS and are only shown when they are ≥ 80%for SH-aLRT and ≥ 95% for UF-BS.
(PDF)

S3 Fig Plot of the first two axes from a principal component analysis (PCA). Unlinked SNPs were randomly subsampled to provide an indication of confidence in the data, with these replicates represented by opaque shading around each data point. SN = Snares, CI = Chatham Islands and AK = Auckland Islands.
(PDF)

S4 Fig Plot of the second two axes from a principal component analysis (PCA). Unlinked SNPs were randomly subsampled to provide an indication of confidence in the data, with these replicates represented by opaque shading around each data point. SN = Snares, CI = Chatham Islands and AK = Auckland Islands.
(PDF)

S1 Table Details of samples used for this study. (DOCX)

S2 Table Fairy (*Pachyptila turtur*) and fulmar prion (*P*. *crassirostris*) samples that failed to amplify. (DOCX)

S3 Table Summary of ddRADseq data. For samples included in the final dataset ‘Loci assembled’ is for the final clustering. For loci not included in the final dataset owing to low numbers of loci and/or duplicates ‘Loci assembled’ is from the preliminary assembly.
(DOCX)

S4 Table Analysis of molecular variance (AMOVA) among fairy and fulmar prions based on mitochondrial COI sequences. Individuals are grouped by species or by island group.
(DOCX)
