# Correction: Genomic analyses of fairy and fulmar prions (Procellariidae: *Pachyptila* spp.) reveals parallel evolution of bill morphology, and multiple species

**DOI:** 10.1371/journal.pone.0315613

**Published:** 2024-12-06

**Authors:** Lara D. Shepherd, Colin M. Miskelly, Mariana Bulgarella, Alan J. D. Tennyson

The dashed line is missing in Fig 3. Please see the correct Fig 3 here.

**Fig 3:**
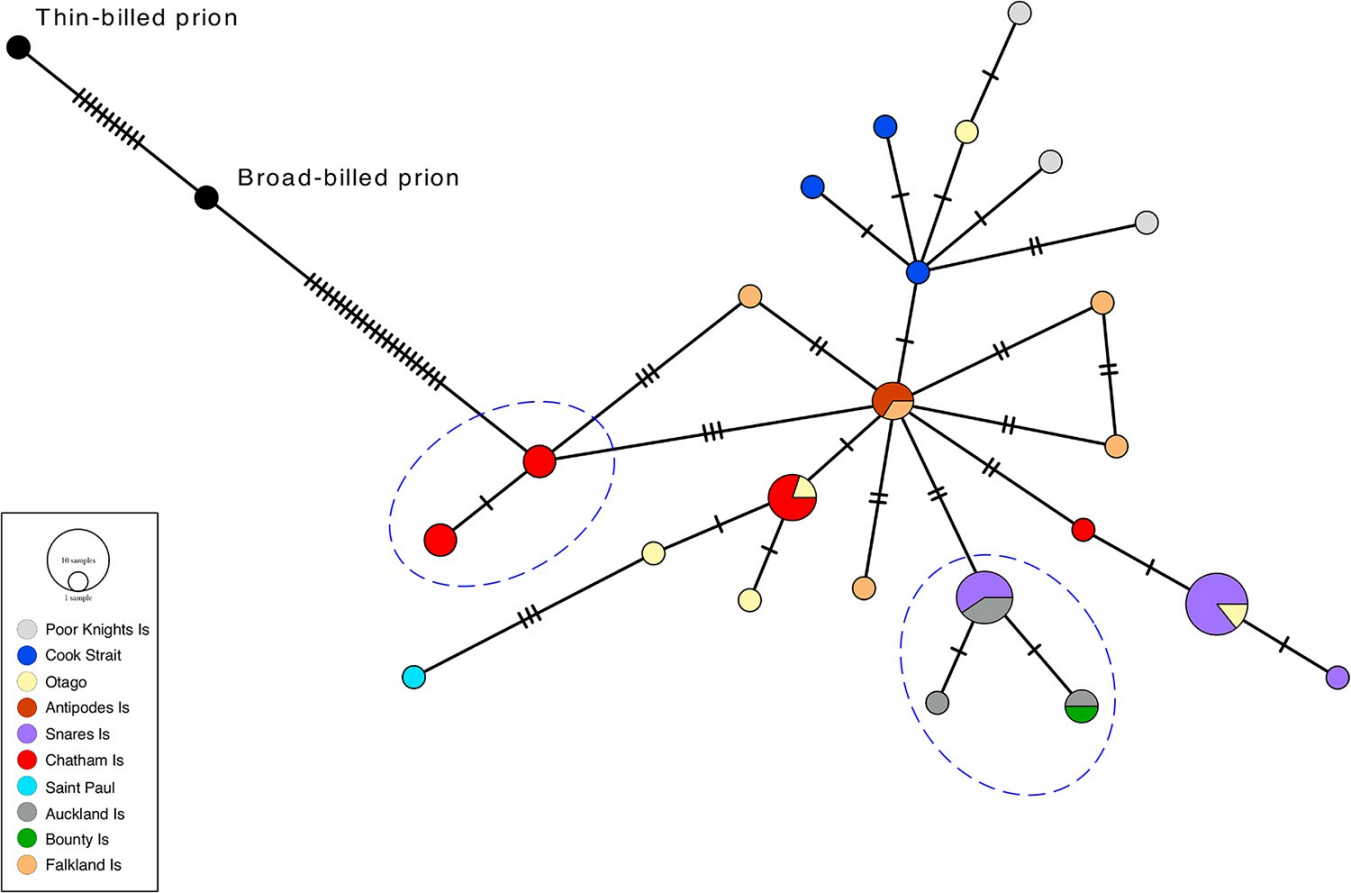
Cytochrome *b* median joining network of fairy and fulmar prions with colour-coded sampling localities.

Fulmar prion samples are encompassed by a dashed line. The size of each circle is proportional to haplotype frequency. Hatch marks represent additional mutational steps separating haplotypes.
