# Supplementary figures and images for: Genomic analyses of fairy and fulmar prions (Procellariidae: Pachyptila spp.) reveals parallel evolution of bill morphology, and multiple species
Source: PLoS One. 2022 Sep 27;17(9):e0275102. doi: 10.1371/journal.pone.0275102 (PMC9514608; doi:10.1371/journal.pone.0275102)

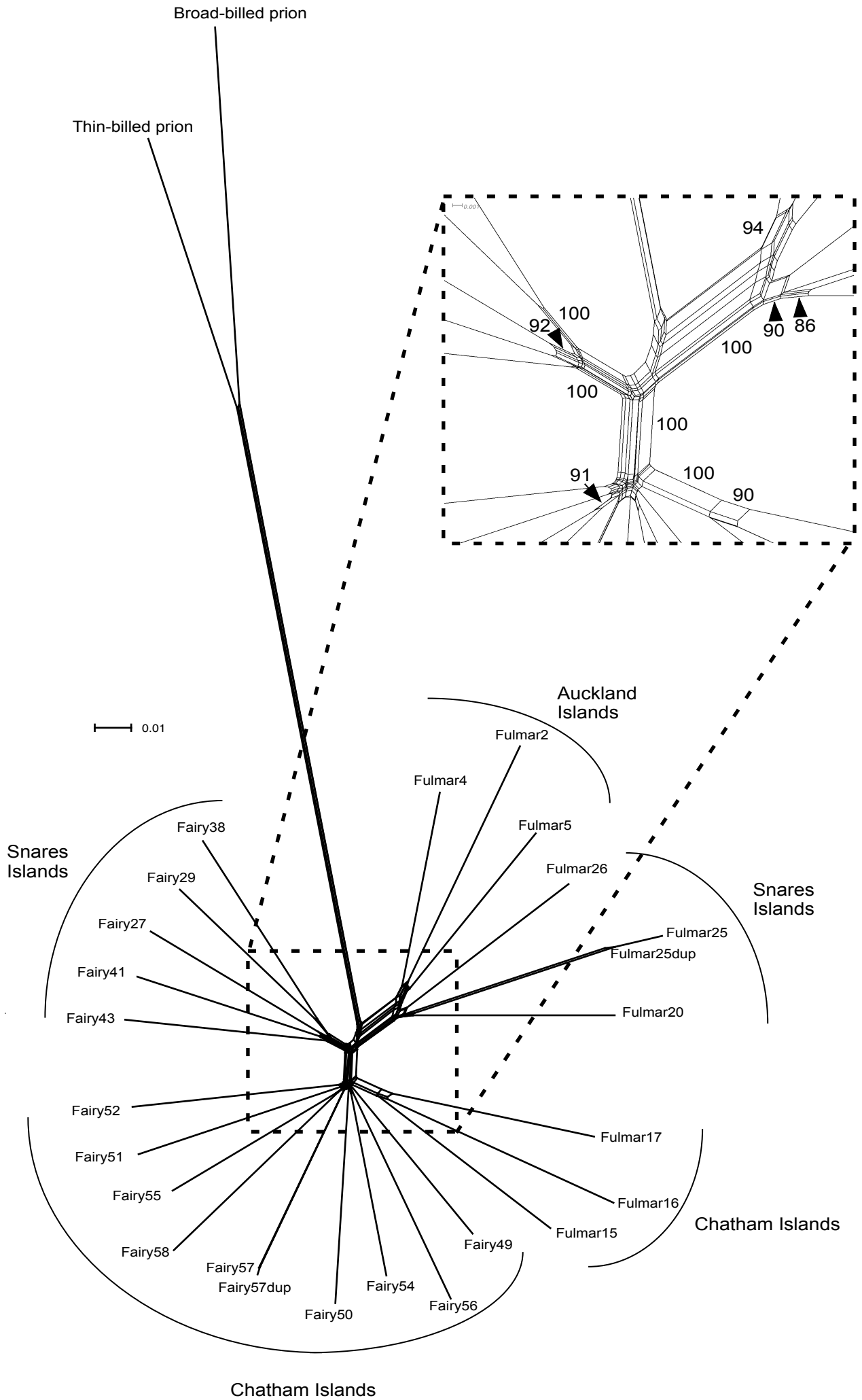

Supplement: S1 Fig — Bootstrap support values over 80% are shown. Duplicates are indicated by ‘dup’ after sequence name and were combined for subsequent analyses. (PDF) [file pone.0275102.s001.pdf]

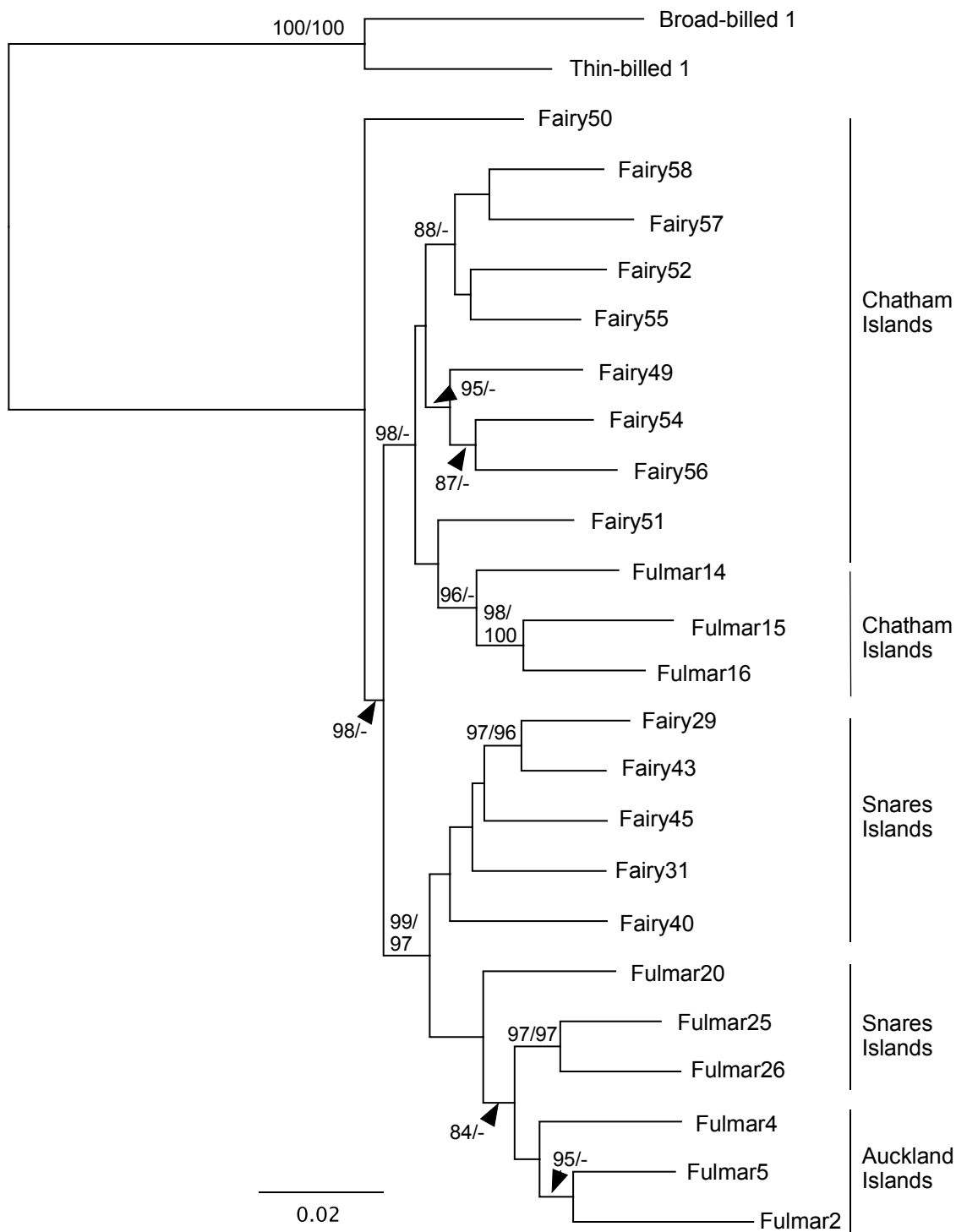

Supplement: S2 Fig — Branch support values are given in the order SH-aLRT/UF-BS and are only shown when they are ≥ 80%for SH-aLRT and ≥ 95% for UF-BS. (PDF) [file pone.0275102.s002.pdf]

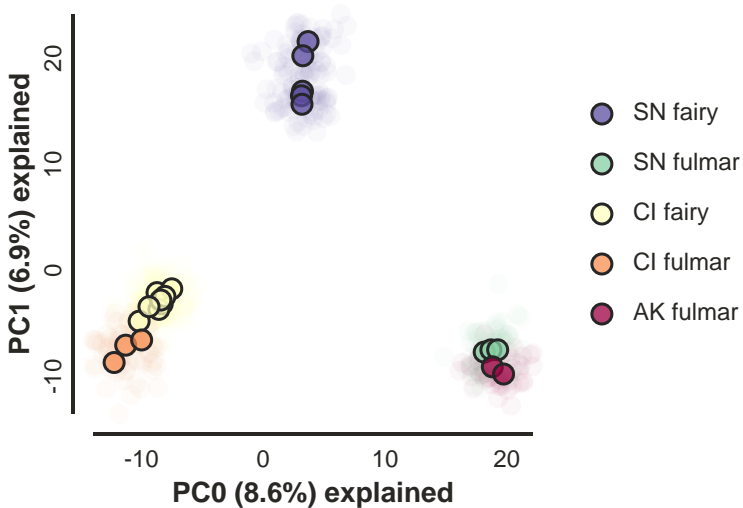

Supplement: S3 Fig — Unlinked SNPs were randomly subsampled to provide an indication of confidence in the data, with these replicates represented by opaque shading around each data point. SN = Snares, CI = Chatham Islands and AK = Auckland Islands. (PDF) [file pone.0275102.s003.pdf]

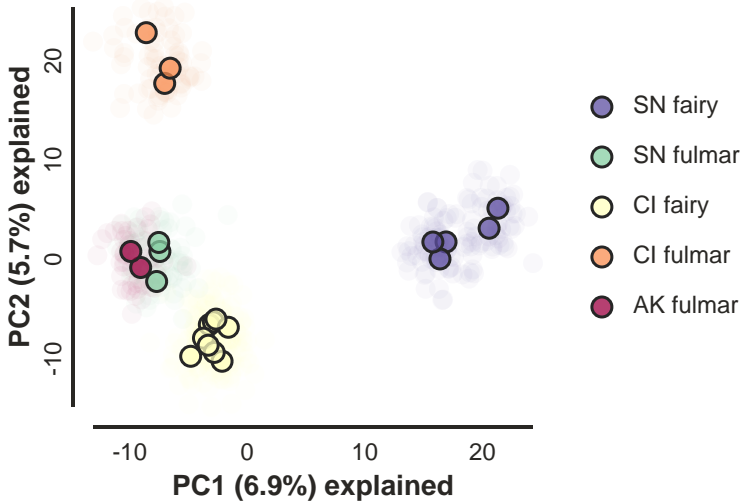

Supplement: S4 Fig — Unlinked SNPs were randomly subsampled to provide an indication of confidence in the data, with these replicates represented by opaque shading around each data point. SN = Snares, CI = Chatham Islands and AK = Auckland Islands. (PDF) [file pone.0275102.s004.pdf]
