# Supplementary material for: Genomic analyses of fairy and fulmar prions (Procellariidae: Pachyptila spp.) reveals parallel evolution of bill morphology, and multiple species
Source: PLoS One. 2022 Sep 27;17(9):e0275102. doi: 10.1371/journal.pone.0275102 (PMC9514608; doi:10.1371/journal.pone.0275102)
Supplement: S1 Table — (DOCX) [file pone.0275102.s005.docx]

**S1 Table. Details of samples used for this study.**

| Species | Sample | Location | Band number/  registration number/  collector’s number | Collector | Sample type | GenBank Accession numbers | | ddRADseq |
| --- | --- | --- | --- | --- | --- | --- | --- | --- |
|  |  |  |  |  |  | COI | Cyt *b* |  |
| *Pachyptila turtur* | Fairy1 | Poor Knights Is., NZ | D-203520 | A. Tennyson | Feathers | OM212731 | OM240603 |  |
|  | Fairy2 | Poor Knights Is., NZ | D-203527 | A. Tennyson | Feathers | OM212732 | OM240630 |  |
|  | Fairy3 | Poor Knights Is., NZ | D-203517 | A. Tennyson | Feathers | OM212733 | OM240606 |  |
|  | Fairy4 | Mana I., Cook Strait, NZ | D-182989 | C. Miskelly | Blood | OM212734 | OM240605 |  |
|  | Fairy5 | Mana I., Cook Strait, NZ | D-147781 | C. Miskelly | Blood | MZ268116 | MZ268100 |  |
|  | Fairy6 | Mana I., Cook Strait, NZ | D-176764 | C. Miskelly | Blood | OM212735 |  |  |
|  | Fairy7 | Mana I., Cook Strait, NZ | D-180569 | C. Miskelly | Blood | OM212736 | MZ054169 |  |
|  | Fairy8 | Mana I., Cook Strait, NZ | D-180558 | C. Miskelly | Blood | OM212738 |  |  |
|  | Fairy9 | Mana I., Cook Strait, NZ | D-180598 | C. Miskelly | Blood | OM212739 |  |  |
|  | Fairy10 | Mana I., Cook Strait, NZ | D-180644 | C. Miskelly | Blood | OM212740 |  |  |
|  | Fairy11 | Mana I., Cook Strait, NZ | D-180406 | C. Miskelly | Blood | OM212741 |  |  |
|  | Fairy12 | St Clair, Dunedin, Otago NZ |  | G. Loh | Tissue | OM212725 |  |  |
|  | Fairy13 | St Clair, Dunedin, Otago, NZ |  | G. Loh | Tissue | OM212746 | OM240612 | Y^^^ |
|  | Fairy14 | St Clair, Dunedin, Otago, NZ |  | G. Loh | Tissue | OM212748 |  | Y^^^ |
|  | Fairy15 | South Westland, NZ | NMNZ OR.029536* | Department of Conservation | Tissue | OM212743 |  |  |
|  | Fairy16 | Herekopare, Southland, NZ | NMNZ OR.030907* | Z. Smith | Tissue | OM212716 | OM240596 |  |
|  | Fairy17 | Herekopare, Southland, NZ | NMNZ OR.030908* | Z. Smith | Tissue | OM212717 | OM240614 |  |
|  | Fairy18 | Antipodes Is. | NMNZ OR.010441* | R.A. Falla | Tissue | OM212727 | OM240609 |  |
|  | Fairy19 | Antipodes Is. | NMNZ OR.010442* | R.A. Falla | Tissue | OM212728 |  |  |
|  | Fairy20 | Antipodes Is. | NMNZ OR.010443* | R.A. Falla | Tissue | OM212730 | OM240610 |  |
|  | Fairy21 | Antipodes Is. | SP520 | E. Sommer | - | MK262321 |  |  |
|  | Fairy22 | North East I., Snares Is. | NMNZ OR.O27217* | A. Tennyson | Tissue | OM212737 |  |  |
|  | Fairy23 | North East I., Snares Is. | NMNZ OR.027258* | A. Tennyson | Tissue | OM212742 |  |  |
|  | Fairy24 | North East I., Snares Is. | D-100964 | C. Miskelly, A. Tennyson | Blood | OM212756 | OM240626 |  |
|  | Fairy25 | North East I., Snares Is. | D-100965 | C. Miskelly, A. Tennyson | Blood | OM212757 |  |  |
|  | Fairy26 | North East I., Snares Is. | D-100966 | C. Miskelly, A. Tennyson | Blood | OM212758 |  | Y^#^ |
|  | Fairy27 | North East I., Snares Is. | D-100967 | C. Miskelly, A. Tennyson | Blood | OM212759 | OM240627 | Y |
|  | Fairy28 | North East I., Snares Is. | D-100968 | C. Miskelly, A. Tennyson | Blood | OM212760 | OM240600 |  |
|  | Fairy29 | North East I., Snares Is. | D-100970 | C. Miskelly, A. Tennyson | Blood | OM212761 |  | Y |
|  | Fairy30 | North East I., Snares Is. | D-100971 | C. Miskelly, A. Tennyson | Blood | OM212762 | OM240601 |  |
|  | Fairy31 | North East I., Snares Is. | D-100972 | C. Miskelly, A. Tennyson | Blood | OM212763 | OM240615 |  |
|  | Fairy32 | North East I., Snares Is. | D-100973 | C. Miskelly, A. Tennyson | Blood | OM212764 |  | Y^^^ |
|  | Fairy33 | North East I., Snares Is. | D-100974 | C. Miskelly, A. Tennyson | Blood | OM212765 |  |  |
|  | Fairy34 | North East I., Snares Is. | D-100975 | C. Miskelly, A. Tennyson | Blood | OM212766 |  |  |
|  | Fairy35 | North East I., Snares Is. | D-100976 | C. Miskelly, A. Tennyson | Blood | OM212767 |  |  |
|  | Fairy36 | North East I., Snares Is. | D-100977 | C. Miskelly, A. Tennyson | Blood | - |  |  |
|  | Fairy37 | North East I., Snares Is. | D-100978 | C. Miskelly, A. Tennyson | Blood | OM212768 |  |  |
|  | Fairy38 | North East I., Snares Is. | D-100969 | C. Miskelly, A. Tennyson | Blood | OM212769 | OM240602 | Y |
|  | Fairy39 | North East I., Snares Is. | D-100979 | C. Miskelly, A. Tennyson | Blood | OM212770 |  |  |
|  | Fairy40 | North East I., Snares Is. | D-100980 | C. Miskelly, A. Tennyson | Blood | OM212771 |  |  |
|  | Fairy41 | North East I., Snares Is. | D-100981 | C. Miskelly, A. Tennyson | Blood | OM212772 | OM240628 | Y |
|  | Fairy42 | North East I., Snares Is. | D-100982 | C. Miskelly, A. Tennyson | Blood | OM212773 | OM240604 |  |
|  | Fairy43 | North East I., Snares Is. | D-100984 | C. Miskelly, A. Tennyson | Blood | OM212774 |  | Y |
|  | Fairy44 | Sisters Is., Chatham Is. | NMNZ OR.019027* | C. Robertson | Tissue | OM212726 |  |  |
|  | Fairy45 | Sisters Is., Chatham Is. | NMNZ OR.17689* | C. Robertson | Tissue | OM212753 |  |  |
|  | Fairy46 | Chatham Is. | NMNZ OR.17692* | B. Bell | Tissue | OM212754 |  |  |
|  | Fairy47 | Sisters Is., Chatham Is. | NMNZ OR.017688* | C. Robertson | Tissue | OM212755 |  |  |
|  | Fairy48 | Mangere I., Chatham Is. | NMNZ OR.018089* | B. Bell | Tissue | OM212802 |  |  |
|  | Fairy49 | Mangere I., Chatham Is. |  | G. Green | Blood | OM212785 | OM240620 | Y |
|  | Fairy50 | Mangere I., Chatham Is. |  | G. Green | Blood | OM212786 |  | Y |
|  | Fairy51 | Mangere I., Chatham Is. |  | G. Green | Blood | OM212787 |  | Y |
|  | Fairy52 | Mangere I., Chatham Is. |  | G. Green | Blood | OM212788 |  | Y |
|  | Fairy53 | Mangere I., Chatham Is. |  | G. Green | Blood | OM212791 | OM240621 |  |
|  | Fairy54 | Mangere I., Chatham Is. |  | G. Green | Blood | OM212788 |  | Y |
|  | Fairy55 | Mangere I., Chatham Is. |  | G. Green | Blood | OM212789 | OM240618 | Y |
|  | Fairy56 | Mangere I., Chatham Is. |  | G. Green | Blood | OM212793 | OM240622 | Y |
|  | Fairy57 | Mangere I., Chatham Is. |  | G. Green | Blood | OM212790 |  | Y |
|  | Fairy58 | Mangere I., Chatham Is. |  | G. Green | Blood | OM212792 | OM240619 | Y |
|  | Fairy59 | Australia | 1B-70 | - | - | MK262374 |  |  |
|  | Fairy60 | Saint Paul, Indian Ocean | - | P. Quillfeldt | Tissue | KX092012 | OM240629 | Y^^^ |
|  | Fairy61 | Ilot Greak, Kerguelen Is. | NMNZ OR.023072* | H. Weimerskirch | Tissue | OM212723 |  |  |
|  | Fairy62 | Falkland Is. | turtur303 |  | - |  | 1 |  |
|  | Fairy63 | Falkland Is. | turtur304 |  | - |  | 1 |  |
|  | Fairy64 | Falkland Is. | turtur305 |  | - |  | 1 |  |
|  | Fairy65 | Falkland Is. | turtur310 |  | - |  | 1 |  |
|  | Fairy66 | Falkland Is. | turtur321 |  | - |  | 1 |  |
|  | Fairy67^2^ | Heard I. | NMNZ OR.024757* | P. Scofield | Tissue | OM212744 |  |  |
|  | Fairy68^2^ | Heard I. | NMNZ OR.024789* | P. Scofield | Tissue | OM212747 |  |  |
|  | Fairy69 | Heard I. | SP245 | P. Scofield, C. Materia | - | MK262197 |  |  |
| *Pachyptila crassirostris* | Fulmar1 | Ewing I., Auckland Is. | NMNZ OR.17498* | B. Bell | Tissue | OM212718 |  |  |
|  | Fulmar2 | Ewing I., Auckland Is. |  | C. Miskelly, A. Tennyson | Blood | OM212798 | OM240599 | Y |
|  | Fulmar3 | Ewing I., Auckland Is. | NMNZ OR.17503* | B. Bell | Tissue | OM212719 |  |  |
|  | Fulmar4 | Disappointment I., Auckland Is. |  | C. Miskelly | Blood | OM212799 | OM240598 | Y |
|  | Fulmar5 | Disappointment I., Auckland Is. |  | C. Miskelly | Blood | OM212800 |  | Y |
|  | Fulmar6 | Bounty Is. | NMNZ OR.025456* | A. Booth | Tissue | OM212729 | OM240611 |  |
|  | Fulmar7 | Bounty Is. | NMNZ OR.021515* | C. Robertson | Tissue | OM212751 |  |  |
|  | Fulmar8 | Bounty Is. | NMNZ OR.021517* | C. Robertson | Tissue | OM212752 |  |  |
|  | Fulmar9 | Forty-Fours, Chatham Is. | NMNZ OR.018463* | C. Robertson | Tissue | OM212722 |  |  |
|  | Fulmar10 | Forty-Fours, Chatham Is. | NMNZ OR.029279* | P. Scofield | Tissue | OM212724 |  |  |
|  | Fulmar11 | Forty-Fours, Chatham Is. | NMNZ OR.018454* | C. Robertson | Tissue | OM212749 |  |  |
|  | Fulmar12 | Forty-Fours, Chatham Is. | NMNZ OR.018456* | C. Robertson | Tissue | OM212750 |  |  |
|  | Fulmar13 | The Pyramid, Chatham Is. | NMNZ OR.018453* | C. Robertson | Tissue | OM212745 |  |  |
|  | Fulmar14 | The Pyramid, Chatham Is. |  | M. Bell | Tissue | OM212794 | OM240613 | Y^#^ |
|  | Fulmar15 | The Pyramid, Chatham Is. |  | M. Bell | Tissue | OM212795 | OM240623 | Y |
|  | Fulmar16 | The Pyramid, Chatham Is. |  | M. Bell | Tissue | OM212796 | OM240624 | Y |
|  | Fulmar17 | The Pyramid, Chatham Is. |  | M. Bell | Tissue | OM212797 | OM240625 | Y |
|  | Fulmar18 | Western Chain, Snares Is. | NMNZ OR.017554* | C. Fleming | Tissue | OM212720 |  |  |
|  | Fulmar19 | Western Chain, Snares Is. | NMNZ OR.017555* | C. Fleming | Tissue | OM212721 |  |  |
|  | Fulmar20 | Toru, Western Chain, Snares Is. | D-100954 | C. Miskelly, A. Tennyson | Blood | OM212775 |  | Y |
|  | Fulmar21 | Toru, Western Chain, Snares Is. | D-100955 | C. Miskelly, A. Tennyson | Blood | OM212776 | OM240597 | Y^^^ |
|  | Fulmar22 | Toru, Western Chain, Snares Is. | D-100957 | C. Miskelly, A. Tennyson | Blood | OM212777 | OM240607 | Y^#^ |
|  | Fulmar23 | Toru, Western Chain, Snares Is. | D-100958 | C. Miskelly, A. Tennyson | Blood | OM212778 |  | Y^^^ |
|  | Fulmar24 | Toru, Western Chain, Snares Is. | D-100959 | C. Miskelly, A. Tennyson | Blood | OM212779 |  | Y^^^ |
|  | Fulmar25 | Toru, Western Chain, Snares Is. | D-100960 | C. Miskelly, A. Tennyson | Blood | OM212780 |  | Y |
|  | Fulmar26 | Toru, Western Chain, Snares Is. | D-100956 | C. Miskelly, A. Tennyson | Blood | OM212781 | OM240616 | Y |
|  | Fulmar27 | Toru, Western Chain, Snares Is. | D-100961 | C. Miskelly, A. Tennyson | Blood | OM212782 | OM240617 | Y^#^ |
|  | Fulmar28 | Toru, Western Chain, Snares Is. | D-100962 | C. Miskelly, A. Tennyson | Blood | OM212783 | OM240608 |  |
|  | Fulmar29 | Toru, Western Chain, Snares Is. | D-100963 | C. Miskelly, A. Tennyson | Blood | OM212784 |  |  |
| *Pachyptila belcheri* | Thin-billed | East of Whangaehu River mouth,  NZ wreck | NMNZ OR.030913 | P. Frost | Tissue | OM212715 | OM240595 | Y |
| *Pachyptila vittata* | Broad-billed1 | Dusky Sound, Fiordland | Dusky BB1 | C. Miskelly, A. Tennyson | Blood |  |  | Y |
|  | Broad-billed2 | Snares Is. | BB_01Snares | C. Miskelly, A. Tennyson | Blood | OM212714 | OM240594 |  |
|  | Broad-billed3 | Snares Is. | BB_02Snares | C. Miskelly, A. Tennyson | Blood |  |  | Y^#^ |
|  | Broad-billed4 | Dusky Sound, Fiordland, NZ | Dusky2 | C. Miskelly, A. Tennyson | Blood |  |  | Y^^^ |
| *Pachyptila desolata* | Antarctic | East of Whangaehu River mouth,  NZ wreck | NMNZ OR.030914 | P.Frost | Tissue |  |  | Y^#^ |
| *Halobaena caerulea* | BluePetrel | Waihi Beach, NZ wreck | NMNZ OR.030915 | E. Lawton | Tissue |  |  | Y^#^ |

^1^Sequences from supplementary material by [8] (https://academic.oup.com/mbe/article/36/8/1671/5480301#supplementary-data)

^2^Previously considered to be fulmar prions (e.g., [12]) but we consider them to be fairy prions.

* sampled from a museum skin and processed in an ancient DNA laboratory.

^ ddRADseq samples excluded prior to analysis owing to low numbers of reads.

# ddRADseq samples excluded from later analyses owing to low number of loci in the final assembly.
