# Supplementary material for: Genomic analyses of fairy and fulmar prions (Procellariidae: Pachyptila spp.) reveals parallel evolution of bill morphology, and multiple species
Source: PLoS One. 2022 Sep 27;17(9):e0275102. doi: 10.1371/journal.pone.0275102 (PMC9514608; doi:10.1371/journal.pone.0275102)
Supplement: S2 Table — (DOCX) [file pone.0275102.s006.docx]

**S2 Table. Fairy (*Pachyptila turtur*) and fulmar prions (*P. crassirostris*) samples that failed to amplify.**

| Species | Location | Voucher | Sample type | Collector | Date |
| --- | --- | --- | --- | --- | --- |
| *Pachyptila turtur* | North Trio I., Cook Strait | NMNZ OR.010317 | Tissue | W. Daubin | 1950 |
|  | Poor Knights Is. | NMNZ OR.015055 | Tissue | P. Harper | 1964 |
|  | Heard I. | NMNZ OR.017288 | Tissue | R. Falla | 1929 |
|  | Heard I. | NMNZ OR.010477 | Tissue | R. Falla | 1929 |
|  | South Georgia | NMNZ OR.027022 | Tissue | I. Hunter | 1980 |
|  | South Georgia | NMNZ OR.027019 | Tissue | I. Hunter | 1980 |
|  | South Georgia | NMNZ OR.027020 | Tissue | I. Hunter | 1980 |
|  | South Georgia | NMNZ OR.027021 | Tissue | I. Hunter | 1980 |
|  | Crozet Is. | NMNZ OR.023091 | Tissue | J. Mougin | 1974 |
|  | Crozet Is. | NMNZ OR.023090 | Tissue | J. Mougin | 1974 |
|  | Crozet Is. | NMNZ OR.023095 | Tissue | J. Provost | 1969 |
|  | Crozet Is. | NMNZ OR.023094 | Tissue | J. Mougin, Despin, Segonzac | 1970 |
|  | Crozet Is. | NMNZ OR.023092 | Tissue | J. Mougin | 1974 |
|  | Crozet Is. | NMNZ OR.023093 | Tissue | J. Mougin, Despin, Segonzac | 1970 |
|  | Antipodes Is. | NMNZ OR.021412 | Tissue | B. Bell | 1978 |
|  | Campbell I. | NMNZ OR.015798 | Tissue | D. Paull | 1970 |
|  | Chatham Is. | NMNZ OR.017687 | Tissue | C. Robertson | 1973 |
|  | Amsterdam I. | NMNZ S.034801 | Bone | J. Martinez | 1994 |
|  | Amsterdam I. | NMNZ S.034592.1 | Bone | J. Martinez | 1994 |
| *Pachyptila crassirostris* | Auckland Is. | NMNZ OR.017500 | Tissue | B. Bell | 1973 |
|  | Auckland Is. | NMNZ OR.010474 | Tissue | R. Falla | 1943 |
