# Supplementary material for: Genomic analyses of fairy and fulmar prions (Procellariidae: Pachyptila spp.) reveals parallel evolution of bill morphology, and multiple species
Source: PLoS One. 2022 Sep 27;17(9):e0275102. doi: 10.1371/journal.pone.0275102 (PMC9514608; doi:10.1371/journal.pone.0275102)
Supplement: S3 Table — For samples included in the final dataset ‘Loci assembled’ is for the final clustering. For loci not included in the final dataset owing to low numbers of loci and/or duplicates ‘Loci assembled’ is from the preliminary assembly. (DOCX) [file pone.0275102.s007.docx]

**S3 Table. Summary of ddRADseq data.** For samples included in the final dataset loci assembled is for the final clustering. For loci not included in the final dataset owing to low numbers of loci and/or duplicates loci assembled is from the preliminary assembly.

| Species | Sample | Number of raw reads | Clusters | Loci assembled | Average depth* |
| --- | --- | --- | --- | --- | --- |
| *Pachyptila turtur* | Fairy26# | 1035814 | 616312 | 1452 | 7.94 |
|  | Fairy27 | 7798314 | 1946410 | 9751 | 15.70 |
|  | Fairy29 | 3442253 | 862090 | 9743 | 11.54 |
|  | Fairy32^ | 217916 |  |  |  |
|  | Fairy38 | 2905863 | 881098 | 9618 | 10.52 |
|  | Fairy41 | 4668927 | 1097121 | 9770 | 13.18 |
|  | Fairy43 | 5035474 | 1085435 | 9774 | 13.70 |
|  | Fairy49 | 5686147 | 1346804 | 9778 | 13.63 |
|  | Fairy51 | 4575073 | 1205321 | 9761 | 11.52 |
|  | Fairy52 | 9271243 | 2359713 | 9766 | 15.58 |
|  | Fairy50 | 3328763 | 1099713 | 9612 | 9.78 |
|  | Fairy54 | 9762932 | 1834710 | 9769 | 18.26 |
|  | Fairy55 | 6484944 | 1394442 | 9768 | 14.40 |
|  | Fairy56 | 2797131 | 986931 | 9471 | 9.21 |
|  | Fairy57 | 5458339 | 1356159 | 11050 | 12.66 |
|  | Fairy57dup | 4990289 | 1960495 | 9770 | 18.52 |
|  | Fairy58 | 10632992 | 1398488 | 9755 | 11.65 |
|  | Fairy13^ | 24719 |  |  |  |
|  | Fairy14^ | 212187 |  |  |  |
|  | Fairy60^ | 90172 |  |  |  |
| *P. crassirostris* | Fulmar2 | 1810936 | 994830 | 3449 | 7.96 |
|  | Fulmar4 | 3645745 | 757407 | 9742 | 12.21 |
|  | Fulmar4dup# | 887098 | 538121 | 982 | 8.33 |
|  | Fulmar5 | 5468621 | 1271201 | 9766 | 13.69 |
|  | Fulmar5dup# | 1441185 | 803703 | 2582 | 8.03 |
|  | Fulmar14# | 1009503 | 614346 | 1147 | 7.88 |
|  | Fulmar15 | 3533511 | 1013866 | 9721 | 11.20 |
|  | Fulmar16 | 2425013 | 845530 | 9468 | 9.50 |
|  | Fulmar17 | 4818535 | 1346796 | 9743 | 12.45 |
|  | Fulmar20 | 6446535 | 1601652 | 9756 | 14.44 |
|  | Fulmar21^ | 140345 |  |  |  |
|  | Fulmar22# | 879072 | 524280 | 953 | 8.16 |
|  | Fulmar23^ | 538819 |  |  |  |
|  | Fulmar24^ | 458942 |  |  |  |
|  | Fulmar25 | 6367525 | 1566105 | 9767 | 14.22 |
|  | Fulmar25dup | 1725452 | 638730 | 8987 | 9.00 |
|  | Fulmar26 | 7023277 | 1445022 | 9770 | 15.92 |
|  | Fulmar27# | 772163 | 465477 | 897 | 8.29 |
| *P. belcheri* | Thin-billed | 2327832 | 1032273 | 9656 | 10.41 |
| *P. vittata* | Broad-billed1 | 2885168 | 732342 | 9672 | 10.92 |
|  | Broad-billed3# | 946199 | 559938 | 1125 | 8.39 |
|  | Broad-billed4# | 1042988 | 624241 | 1259 | 8.11 |
| *P. salvinii* | Salvins# | 1418999 | 828419 | 2556 | 7.86 |
| *Halobaena caerulea* | BluePetrel^ | 678536 |  |  |  |

*after excluding loci with depth <6

^excluded prior to analyses owing to low numbers of reads

#excluded from analyses after preliminary assembly owing to low number of loci

dup = sample performed in duplicate
