# Supplementary material for: Genomic analyses of fairy and fulmar prions (Procellariidae: Pachyptila spp.) reveals parallel evolution of bill morphology, and multiple species
Source: PLoS One. 2022 Sep 27;17(9):e0275102. doi: 10.1371/journal.pone.0275102 (PMC9514608; doi:10.1371/journal.pone.0275102)
Supplement: S4 Table — Individuals are grouped by species or by island group. (DOCX) [file pone.0275102.s008.docx]

**S4 Table. Analysis of molecular variance (AMOVA) among fairy and fulmar prions based on mitochondrial COI sequences.** Individuals are grouped by species or by island group.

| Grouping | Source of variation | d.f. | Variation (%) | Fixation indices |
| --- | --- | --- | --- | --- |
| Species | Among groups | 1 | 0.56 | F_CT_ = 0.01 |
|  | Among populations | 13 | 47.66 | F_SC_ = 0.48* |
|  | Among groups | 80 | 51.78 | F_ST_ = 0.48* |
| Geography | Among groups | 10 | 41.61 | F_CT_ = 0.42* |
|  | Among populations | 5 | 8.85 | F_SC_ = 0.14* |
|  | Among groups | 79 | 49.54 | F_ST_ = 0.50* |
| SAMOVA grouping (K=3) | Among groups | 2 | 49.51 | F_CT_ = 0.49* |
|  | Among populations | 13 | 7.12 | F_SC_ = 0.14 |
|  | Among groups | 79 | 43.38 | F_ST_ = 0.57* |

* Significant values (P < 0.05)
